# Use and abuse of dietary supplements in persons with diabetes

**DOI:** 10.1038/s41387-020-0117-6

**Published:** 2020-04-27

**Authors:** Bridget A. Hannon, William D. Fairfield, Bryan Adams, Theodore Kyle, Mason Crow, Diana M. Thomas

**Affiliations:** 1 Division of Nutritional Sciences, University of Illinois at Urbana-Champaign, Urbana, IL USA; 2 Department of Kinesiology and Community Health, University of Illinois at Urbana-Champaign, Urbana, IL USA; 3 Department of Mathematical Sciences, United States Military Academy, West Point, NY USA; 4 ConscienHealth, Country Club Dr, Pittsburgh, PA USA

**Keywords:** Obesity, Diagnostics

## Abstract

The dietary supplement industry has estimated sales of over $30 billion in the US and over $100 billion globally. Many consumers believe that dietary supplements are safer and possibly more effective than drugs to treat diabetes. The sheer volume of the literature in this space makes compiling them into one review challenging, so much so that primarily narrative reviews currently exist. By applying the interactive database supplied by the Office of Dietary Supplements at the National Institutes of Health, we identified the top 100 ingredients that appeared most often in dietary supplement products. One-hundred different keyword searches using the ingredient name and the word diabetes were performed using a program developed to automatically scrape PubMed. Each search was retained in a separate Excel spreadsheet, which was then reviewed for inclusion or exclusion. The studies that met the inclusion criteria were evaluated for effect of reducing and controlling diabetes. The PubMed scrape resulted in 6217 studies. For each keyword search only the most recent 100 were retained, which refined the total to 1823 studies. Of these 425 met the screening criteria. The ingredients, fiber, selenium and zinc had the most studies associated with improvement in diabetes. Several popular supplement ingredients (phosphorus, pantothenic acid, calcium, magnesium, glutamine, isoleucine, tyrosine, choline, and creatine monohydrate) did not result in any studies meeting our screening criteria. Our study demonstrates how to automate reviews to filter and collapse literature in content areas that have an enormous volume of studies. The aggregated set of studies suggest there is little clinical evidence for the use of dietary supplements to reduce or control diabetes.

## Introduction

Dietary supplements comprise a vibrant market in the United States (US) and around the world. Industry estimates suggest that sales of such products for all indications amount to more than $30 billion in the US^1^, and estimates for global sales exceed $100 billion^2^. Supplement use remains popular among consumers, despite the lack of evidence for many popular supplements on the market. Consumers may use supplements in hopes of improving or maintaining their health, to correct a dietary deficiency, or more therapeutically for a specific health condition.

US regulatory oversight for dietary supplements is distinctly different from the framework for pharmaceuticals^3^. Makers of a new drug must submit evidence for safety and efficacy to the US Food and Drug Administration (FDA) for prior review and approval before it can be used on the market. The same standard does not apply to dietary supplements. Dietary supplements, by law, are not intended to diagnose, treat, prevent, or cure any disease. Therefore, FDA-approved evidence of safety and efficacy for supplements is not needed prior to their appearance on the market. Likewise, there are no regulations regarding the validity of products claims that can be made, with the exception that claims cannot state that a supplement may treat, prevent, or cure a disease^4^. For example, a supplement maker cannot claim that their product is intended for diabetes treatment. They can, however, claim that a product helps to maintain healthy blood sugar levels, so long as they are not suggesting that the product can help a consumer with elevated blood sugar. In practice, delineating these distinctions can be very difficult to make for consumers to interpret the potential consequences of a supplement^5^.

Another difference between dietary supplements and pharmaceuticals is the regulation of their manufacturing. Both drugs and supplements must be manufactured according to good manufacturing practices (GMPs). However, supplements must meet the different and generally lower standard for GMPs that applies to drugs^6^. The stringent requirements for active drug ingredients do not apply to supplements. Because a new supplement product is not subject to FDA approval, an FDA manufacturing inspection is not required, and the FDA does not routinely analyze the content of dietary supplements.

Responsibility for enforcement regarding potentially deceptive claims about dietary supplements falls principally to the Federal Trade Commission (FTC). But the volume of potential violations far exceeds the capacity of FTC to take enforcement actions^3^. Claims of potential efficacy of dietary supplements for preventing or controlling diabetes mellitus are common and may easily be found from sources that consumers might consider to be authoritative. For instance, Healthline lists 10 supplements to help lower blood sugar^7^.

What is the evidence for supplements as a benefit for patients with diabetes? The American Diabetes Association Standards of Medical Care in Diabetes states that there is insufficient evidence to support a benefit from supplements for patients with diabetes who have no underlying deficiencies. Thus, they are not recommended for glycemic control^8^. Despite this, there have been several narrative reviews highlighting the potential benefits of various supplements for diabetes-related outcomes^9–11^. However, many of the studies that have been reviewed may not be of appropriate design or quality to provide strong evidence for or against supplement use.

Examining reviews of supplements and their benefits is challenging, because searching directly using key words, such as “diabetes” and “dietary supplement” within pubmed.gov or other medically related search engines results in an unmanageable set of publications that are incomplete and difficult to organize. In addition, the active ingredients within a supplement serve as the basis of many studies and would not be captured in the search results. The objective of this review was to identify supplement ingredients commonly used for diabetes management and evaluate the scientific evidence supporting their use in patients with type 1 (T1D) and type 2 (T2D) diabetes mellitus.

## Methods

### Dietary supplement ingredient list

The Office of Dietary Supplements (ODS) at the National Institutes of Health developed a searchable database, the Dietary Supplement Label Database (DSLD) available at the URL https://dsld.nlm.nih.gov/dsld/^12^. The database houses information from approximately 76,000 dietary supplement products commercially available in the US. Within the advanced search, we selected an option to search “by Label Statement or Health Claims contains”. In this we input the key word diabetes. The ingredient list for the resulting search was downloaded as a.csv file and retained. Code was written in the statistical software R (**R** Core Team (2013)) to count the number of times the ingredient appeared in a product.

The ingredients were sorted in descending order by the number of times the ingredient appeared on a product label. Spurious information that were not specific ingredients, like “total calories” were removed from the list. From the remainder of the list the top 100 ingredients based on how often they appeared in products were retained (see Supplemental Materials).

### Web-scraping program developed to search pubmed.gov

We then used the RSelenium^13^ package to create a program in the statistical software package R (**R** Core Team (2013)) to automatically collect or “scrape” information from listings and abstracts in the PubMed database pertaining to both the ingredients on our ingredient lists and diabetes research. The program automatically combined each ingredient in the final retained database derived from the ODS website with “(ingredient) AND Diabetes” when searched on PubMed, e.g. “(Potassium) AND Diabetes.” Additional searches were not made for diabetes comorbidities or T1D versus T2D. The program then automated the search with these key words using PubMed built in filters to filter articles that contained an abstract and gathered pertinent article information from PubMed for up to the 100 most recent articles. The information the program scraped included the title, author, journal, year, URL, DOI, and abstract. Additionally, the program removed any redundant articles that may have existed on PubMed. The search also allowed for flagging of certain words in the title and abstract. The following phrases were flagged and counted for each ingredient: cohort, observational, randomized control trial (RCT), meta-analysis, systematic review, clinical trial, HbA1c, fasting glucose, and insulin. Results were automatically retained in separate spreadsheets by ingredient name.

### Article screening

Two members of the research team (BAH and WDF) screened the 100 most recent abstracts from the included ingredients. The following information was extracted from every article: was the study conducted in individuals with diabetes (yes/no), was the study conducted in an animal model (yes/no), does the study meet inclusion criteria (yes/no). Inclusion criteria involved a study examining the role of the individual ingredient on outcomes related to diabetes (including glucose, insulin, HbA1c, diabetes-related complications, etc.) in animal or human subjects. Exclusion criteria involved studies examining the effects of a multi-nutrient supplement or co-nutrients, cross-sectional or observational studies, studies relying on self-reported dietary or supplement intake, studies that included caloric restriction, or studies with an outcome not related to diabetes. If an abstract was found to meet inclusion criteria, the following information was extracted: study type (RCT, single-arm trial, crossover, meta-analysis, narrative review, etc.), outcome, and whether results support the use of that ingredient for T1D or T2D. If a study did not meet inclusion criteria, the reason for exclusion was noted.

### Cross checking and discrepancy resolution

After the first pass of article screening, four members of the research team (BAH, WDF, DMT, MWC) cross-checked the initial abstract screen. Data extracted from this step included confirmation that the study examines the effect of a supplement on diabetes-related outcomes in either a human or animal model and if a discrepancy is present between the original screener and the cross-checker. If there was a discrepancy identified, notes were retained on the reason for the discrepancy. Finally, all discrepancies were reviewed by DMT for validity. Studies that had been flagged as a discrepancy were reviewed and discarded or retained after cross-checking retainment criteria.

### Evidence grading

Each included study was assigned to one of the following evidence grades: 1: meta-analysis of human RCTs, 2: human or animal RCT, 3: human or animal single arm trial, 4: narrative review, position statement, or case report^14^.

## Results

**Fig. 1:**
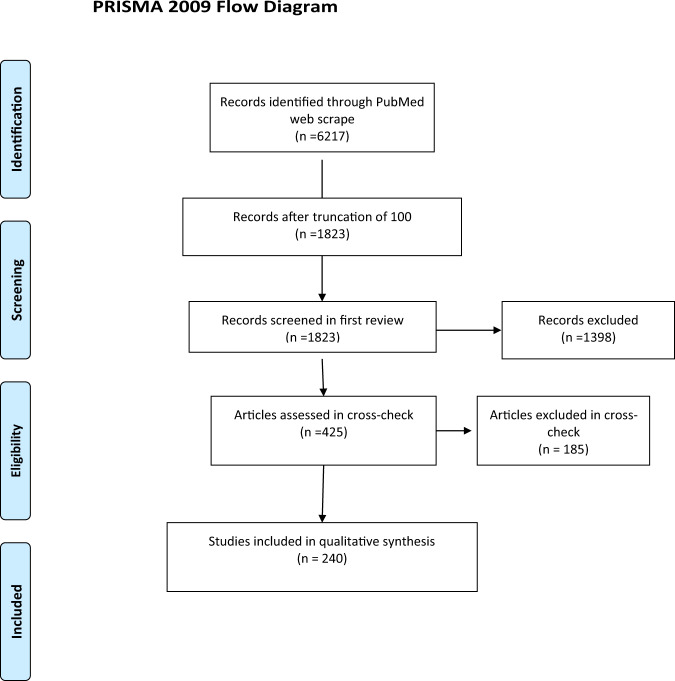
PRISMA Flow Diagram. Article screening process depicted in the preferred reporting items for systematic reviews and meta-analyses (PRISMA) flow chart.

The Preferred Reporting Items for Systematic Reviews and Meta-Analyses (PRISMA) flow chart for article screening is provided in Fig. 1. There were 2086 ingredients on the ODS website included the word diabetes in their label statement or claim. From the 100 most common ingredients, there were 6,217 articles in PubMed found in the searches from each ingredient and the keyword diabetes (Table 1). If ingredients had greater than 100 articles, the 100 most recent were included for screening. From the remaining 1823 abstracts, 425 were retained for full text screening. Many studies were excluded at this phase for evaluation of multinutrient supplements, observational study design, or outcomes not related to diabetes. After the 425 articles were evaluated and discrepancies were resolved, 240 studies remained. Common reasons for exclusion at this stage included the use of multinutrient supplementation, reliance on self-report dietary measures, or outcome not related to diabetes. The 240 included studies examined over 100 different diabetes-related comorbidities, including outcomes related to insulin dynamics (secretion, production, resistance, sensitivity, HOMA-IR, and HOMA-B), glucose metabolism (postprandial, fasting, oral tolerance, two-hour oral tolerance, glycemic response, and HbA1c), and hepatic and pancreatic morphology and function (liver function tests, steatosis, beta cell function, and histology). Other studies examined outcomes related to oxidative stress (antioxidant enzyme activity, antioxidant capacity, endothelial dysfunction, reactive oxidative species formation, glutathione activity, endoplasmic reticulum stress, etc.) or molecular and microbial changes (expression of genes such as *GLUT4*, *NFkB*, *PI3K*, *mTOR*, *TNFα*, *TGFβ*, and *VEGF*, gut microbial diversity, microbial dysbiosis, and concentration of lipopolysaccharide binding protein). Outcomes related to complications of chronic hyperglycemia (advanced glycation end product formation, retinopathy, neuropathy, nephropathy, kidney function, wound healing, vascular function, immune function, etc.) and comorbidities of diabetes (body composition, waist circumference, blood lipid concentrations, blood pressure, bone integrity, C-reactive protein concentrations, incidence of the Metabolic Syndrome, mortality, etc.) were also explored. A final ingredient list with included studies are outlined below and in Table 2. References for all included studies are available in the Supplementary Information.

**Table 1:** Top 100 most common ingredients in the Office of Dietary Supplements database that list “diabetes” on their label or health claim.

Ingredient	Number of Articles in PubMed	Terms extracted from abstracts								
		Cohort	Observational	RCT	Meta-analysis	Systematic review	Clinical trial	HbA1c	Fasting Glucose	Insulin
Protein	2067	4	3	7	2	2	6	9	4	39
“Total Carbohydrate”	493	8	5	14	12	8	20	30	11	52
Calcium	397	10	12	6	4	2	6	3	3	11
Dietary fiber	351	6	4	3	2	7	10	7	4	27
Vitamin E	244	4	4	4	2	0	9	4	4	29
Vitamin C	199	7	3	6	5	4	8	6	3	21
Chromium	184	4	0	6	5	2	8	11	7	52
Zinc	182	1	3	5	8	8	11	12	5	43
Folic acid	166	9	6	6	5	5	4	6	4	11
Magnesium	147	8	3	11	5	4	8	7	5	30
Sodium	140	2	3	3	2	2	7	3	2	19
Selenium	120	4	7	8	5	2	13	3	4	28
Potassium	91	2	2	2	1	3	4	1	1	18
Vitamin B12	88	8	3	2	0	4	7	1	2	14
Phosphorus	51	3	6	3	1	1	1	2	0	6
Vitamin B6	48	5	2	1	0	0	4	3	1	6
Trans fat	45	2	2	0	0	0	2	1	0	15
l-Tyrosine	44	1	0	1	0	1	0	2	1	25
Taurine	42	0	0	0	0	1	1	0	0	15
l-Leucine	41	1	1	0	1	1	2	0	0	23
l-Glutamine	38	0	0	1	0	0	0	3	1	12
Caffeine	28	5	0	0	0	1	0	1	0	9
Choline Bitartrate	23	0	0	0	0	0	0	0	0	4
Niacin	21	2	1	0	0	0	2	0	1	5
Biotin	17	0	0	1	1	1	3	1	1	9
Beta-Alanine	15	0	0	0	0	0	0	2	1	5
Beta-Alanine	15	0	0	0	0	0	0	2	1	5
l-Isoleucine	5	1	0	0	0	0	0	0	0	2
l-Valine	4	1	0	0	0	0	0	0	0	2
Creatine Monohydrate	3	0	0	0	1	0	1	0	0	0
“Strength Matrix”	2	0	0	0	0	0	0	0	0	0
Pantothenic acid	2	0	1	1	0	1	0	0	0	0
Caffeine Anhydrous	0	0	0	0	0	0	0	0	0	0
Agmatine Sulfate	0	0	0	0	0	0	0	0	0	0
*N*-Acetyl-l-Tyrosine	0	0	0	0	0	0	0	0	0	0
Creatine Nitrate	0	0	0	0	0	0	0	0	0	0
l-Citrulline Aspartate	0	0	0	0	0	0	0	0	0	0
Vinpocetine	0	0	0	0	0	0	0	0	0	0

Each ingredient was searched in PubMed using the ingredient name and supplement and506 diabetes. The terms listed above were extracted from the abstract and counted above507 using R.

*RCT* randomized control trial, *HbA1C* hemoglobin A1C.

**Table 2:** Summary table containing number of studies in each study grade category (Meta-analyses = Grade 1, RCT = Grade 2, Single Arm or Cross-over = Grade, narrative review or Case Study = Grade 4), support for diabetes benefit and overall description by supplement ingredient.

Nutrient	Total studies	Meta-analyses	RCT		Single Arm or cross-over	Narrative review or case study	Do results support the use of this supplement for diabetes management? (Yes, No, Mixed)	Brief summary
			Human	Animal				
*Water-soluble vitamins*								
Niacin	3	1	–	2	–	–	Yes (1); No (2)	Meta-analysis of human RCTs concluded that niacin supplementation resulted in increased onset of T2DM cases; animal studies examined outcomes of oxidative stress, glucose tolerance, and insulin sensitivity, with mixed results.
Vitamin B6	4	–	–	3	–	1	Yes (2); No (1); Mixed (1)	Supplementation may improve diabetic neuropathy, fasting glucose, and diabetes-related endothelial dysfunction in mice. Narrative review of human studies concluded no benefit.
Biotin	4	–	–	1	–	3	Yes (2); No (1); Mixed (1)	In animals, biotin was only effective when combined with exercise. Narrative review of human trials concluded that high-dose biotin may improve fasting glucose and glucokinase function.
Folate/Folic Acid	6	3	2	–	1	–	Yes (2); No (1); Mixed (3)	Meta-analyses concluded conflicting findings on folic acid supplementation for fasting glucose and HOMA-IR. Supplementation did not result in improvements in HbA1c or homocysteine, but did improve carotid intima media thickness in adults with MetS and decreased risk of stroke in adults with T2DM when co-administered with ACE inhibitor.
Vitamin B12	5	–	1	–	–	4	Yes (2); No (3)	B12 supplementation may be important in individuals on Metformin, as this drug reduces serum levels of the vitamin. Experimental evidence does not support B12 supplementation for diabetes-related outcomes.
Vitamin C	15	5	4	5	–	1	Yes (6); No (3); Mixed (6)	Meta-analyses concluded that supplementation may improve fasting glucose and diastolic blood pressure, but not HbA1c, in adults with T2DM. Human clinical trials conclude no benefit of supplementation on fasting glucose, blood lipids, or onset of T2DM, but may improve anxiety symptoms. Findings from animal models indicate improvements in antioxidant capacity and T lymphocyte function.
*Fat-soluble vitamins*								
Vitamin E	22	5	8	6	1	1	Yes (12); No (8); Mixed (2)	Meta-analyses found no effect on markers of glucose or insulin dynamics with the exception of one displaying improved HbA1C in subjects with uncontrolled glycemia at baseline and low baseline Vitamin E levels. Improvements from human clinical trials indicated mixed findings. A meta-analysis of animal trials displayed significant improvements in wound healing.
*Minerals*								
Chromium	37	6	7	21	–	3	Yes (17); No (8); Mixed (12)	Meta-analyses display mixed results on glucose control, HbA1C, and TG concentrations. Human and animal trials found mixed results on HbA1C, insulin, and oxidative stress. One meta-analysis found that chromium deficiency was associated with impaired glucose tolerance.
Potassium	1	–	1	–	–	–	Yes (1); No (0)	Single trial in humans concluded improvements in fasting glucose, but not OGTT following supplementation, despite weight gain. weight gain
Selenium	25	2	10	7	2	4	Yes (15); No (5); Mixed (5)	No improvement in risk of diabetes in meta-analyses. Human clinical trials suggest improvements in glucose, insulin, insulin resistance, blood lipids, and inflammation. Animal trials display improvements in anti-oxidant enzyme activity, blood glucose, and insulin sensitivity.
Sodium	7	–	1	5	3	–	Yes (5); No (0); Mixed (2)	In one human trial, GLP-1 was improved but no other measurements related to diabetes. Animal trials display improvements in glucose control, insulin measurements, and body weight.
Zinc	36	4	6	14	1	10	Yes (30); No (4); Mixed (2)	Meta-analyses concluded improvements in fasting glucose, HbA1c, fasting insulin, and markers of diabetic kidney injury. Human RCTs found mixed, but mostly positive, effects of supplementation on reducted progression to diabetes in pre-diabetes, improvements in fasting glucose, OGTT, insulin resistance, and blood lipids. Animal trials suggest zinc’s potential to augment metformin treatment, other positive effects on glucose control, insulin, and oxidative stress.
*Amino acids*								
Beta-Alanine	1	–	–	–	–	1	Yes (1); No (0)	May indirectly improve T2DM complications through increases in intramuscular carnosine concentrations.
Taurine	17	–	–	12	3	2	Yes (9); No (3); Mixed (6)	Human crossover trials found no effect on insulin sensitivity or platelet aggregation. Animal work indicates potential benefit for complications including diabetic retinopathy and endothelial dysfunction, but mixed results for fasting glucose, beta cell function, and glucose tolerance. Narrative reviews cite the potential of taurine yet the lack of clinical trials.
l-Leucine	18	–	1	11	–	6	Yes (11); No (4); Mixed (3)	Only human RCT found no benefit on improvements in HbA1C or insulin sensitivity. Animal studies evidenced improvements in fasting glucose and OGTT, but mixed effects on insulin resistance. Supplementation did not improve pancreatic insulin output. Narrative reviews concluded mixed results on glucose homeostasis.
*Other*								
Caffeine	3	–	1	1	–	1	Yes (0); No (2); Mixed (1)	Human RCT in T1DM patients concluded caffeine may attenuate post-exercise drop in glycemia, but also may result in late-onset hypoglycemia. Animal study showed no benefit on platelet aggregation or ATP signaling.
Dietary fiber	26	1	6	10	1	8	Yes (18); No (2); Mixed (6)	Many different fiber sources were tested. Meta-analysis results show improvements in HbA1C, HOMA-IR, and insulin levels following soluble fiber supplementation. Human RCTs demonstrated benefits of soluble fiber on postprandial and fasting glucose, but not intravenous glucose tolerance. Animal RCTs examined various soluble fibers and prebiotics and demonstrated benefits in body weight, hyperglycemia, hyperinsulinemia, and microbial diversity. Several narrative reviews on prebiotics suggest their benefit for microbial diversity, and improvements in glucose and insulin concentrations.
Protein	4	–	1	1	–	1	Yes (2); No (1); Mixed (1)	Human RCT results in mixed effects on adipokine profiles. Animal trials suggest benefits for insulin sensitivity following beta-conglycinin supplementation; but glucosamine supplementation induced insulin resistance. A narrative review suggests milk proteins may improve postprandial glucose.
Trans fat	6	–	1	4	1	1	Yes (2); No (1); Mixed (2)	CLA supplementation impaired insulin sensitivity in men with obesity, but improved insulin secretion in animal studies, despite other mixed results. Trans-vaccenic acid improved insulin sensitivity in rats.
Totals	240	27	50	103	13	47		

*ACE* angiotensin converting enzyme, *ATP* adenosine triphosphate, *CLA* conjugated linoleic acid, *GLP-1* glucagon-like peptide 1, *HbA1C* hemoglobin A1C, *HOMA-IR* homeostatic model of insulin resistance, *MetS* Metabolic Syndrome, *OGTT* oral glucose tolerance test, *RCT* randomized control trial, *T1DM* type 1 diabetes mellitus, *T2DM* type 2 diabetes mellitus, *TG* triglycerides.

### Water-soluble vitamins

Water-soluble vitamins that had relevant studies included vitamin C^15^, folate/folic acid^6^, vitamin B12^5^, vitamin B6^4^, biotin^4^, and niacin^3^. Meta-analyses examining vitamin C supplementation and diabetes-related outcomes concluded that supplementation may improve fasting blood glucose but not HbA1c in individuals with T2D^15–17^. Findings from human and animal clinical trials were mixed. Three meta-analyses of folate or folic acid supplementation had conflicting findings^18–20^. Relevant studies examining effects of B12 supplementation were limited to individuals taking Metformin, as this drug can deplete serum B12 levels. Human studies on both B6 and biotin were extremely limited, with narrative reviews on both vitamins concluding a lack of evidence for their benefit among T2D patients^21^. One meta-analysis on niacin supplementation was found, which concluded an increased risk of T2D onset following supplementation^22^.

### Fat-soluble vitamins

Vitamin E^22^ was the only fat-soluble vitamin with relevant studies. Included meta-analyses displayed no benefit to measurements of glucose or insulin with the exception of one showing improvements in HbA1C in individuals with uncontrolled glycemia and low serum vitamin E at baseline. Human and animal trials displayed mixed results on fasting glucose, insulin, and markers of inflammation. Results of human clinical trials were not dose dependent, exhibiting variability regardless of dose.

### Minerals

Minerals were the most widely studied category in this review, accounting for 106 studies. Included minerals were chromium^23^, potassium^1^, selenium^24^, sodium^7^, and zinc^25^. Chromium is well studied in relation to diabetes, and one notable review highlighted the potential association between chromium deficiency and hyperglycemia and impaired glucose tolerance^26^. However, results from supplementation trials in both humans and animals were mixed. One study on potassium supplementation was conducted in individuals with prediabetes and concluded that potassium supplementation improved fasting blood glucose despite weight gain, but no significant effects were observed for oral glucose tolerance test or insulin sensitivity.

Selenium accounted for 25 studies with mostly positive results. The two meta-analyses investigated risk of diabetes following supplementation, but concluded no benefit^24,27^. Human clinical trials found improvements in measurements of glucose, insulin, insulin resistance, and blood lipids, and markers of inflammation. Results from animal trials include improvements in anti-oxidant enzyme activity, blood glucose, and insulin sensitivity. However, one review cites the positive correlation between selenium and diabetic risk as well as its hyperglycemic effects in rats^28^.

Evidence on sodium supplementation was unsubstantial. No meta-analyses were included, and the only human clinical trial found an improvement in GLP-1 expression with no improvements on glycemia, insulin, or anthropometric measurements.

Thirty-six studies on zinc supplementation met inclusion criteria, and thirty of those reported positive results. Three meta-analyses found improvements in fasting glucose, HbA1c, and insulin^29–31^. Another found improvements in markers of diabetic kidney injury^29^. Zinc supplementation in a trial of pre-diabetic individuals reduced progression to diabetes along with improvements in fasting glucose, oral glucose tolerance test (OGTT) results, insulin resistance, and blood lipids. Other included human trials display mixed results with many showing improvements in similar markers. One trial in streptozotocin-induced diabetic rats, zinc displayed the potential to augment metformin’s improvements on glucose control^32^. Other animal trials show mostly positive effects of zinc supplementation on glucose control, insulin, and oxidative stress.

### Amino acids

Eighteen studies on leucine, seventeen on taurine, and one on Beta-Alanine supplementation met inclusion criteria. Only one human RCT was found for leucine supplementation, and concluded no effect on glucose or insulin sensitivity^33^. Many potential articles were excluded for examining multiple amino acids in conjunction. Animal studies in leucine supplementation indicated potential benefits for glycemia (fasting glucose, oral glucose tolerance) and pancreatic insulin secretion, but no effect on β-cell development, fasting insulin, or blood lipid concentrations. Narrative reviews highlighted the role of leucine as a potential insulin secretagogue to improve glucose homeostasis, but the mechanism remains unknown. Three human crossover trials were identified for taurine supplementation, two of which concluded no benefit on insulin sensitivity or platelet aggregation^34,35^. The third was conducted in patients with type 1 diabetes, and showed benefits of supplementation for vascular stiffness^36^. Among the 12 animal RCTs reviewed, there were promising results for taurine supplementation on diabetic retinopathy, endothelial dysfunction, insulin sensitivity, and polydipsia/polyuria. There were mixed results regarding beta cell function and glycemia. Narrative reviews stated that taurine may be beneficial for diabetes but cite a lack of clinical evidence.

### Fiber, macronutrients, and caffeine

Twenty-six studies were reviewed on dietary fiber supplementation. One meta-analysis of human RCTs found beneficial effects of soluble fiber supplementation on HbA1c, fasting glucose, and HOMA-IR^25^. Human clinical trials conclude positive results following supplementation of a wide range of fibers, including insoluble fiber, galacto-oligosaccharides (GOS), chicory inulin, and beta-glucan. Animal RCTs concluded beneficial effects of soluble fiber supplementation (from wheat bran extract, GOS, barley, and beta-glucan) on outcomes related to glucose, HbA1c, and microbial diversity). There were mixed effects of supplementation on insulin sensitivity. Narrative reviews highlighted the potential benefits of prebiotics on glycemic and microbial outcomes and soluble fiber for glycemic response, insulin concentrations, and body weight.

Additionally, there were six studies on trans-fat supplementation, four on protein supplementation, and three on caffeine supplementation that met inclusion criteria. The most common trans-fat supplementation was conjugated linoleic acid (CLA), which negatively impacted insulin sensitivity in prediabetic men, despite having beneficial effects on insulin secretion in animals^23^. Trans-vaccenic acid also improved insulin sensitivity and secretion in animals. In humans, protein supplementation had mixed effects on adipokine concentrations, yet improved adiponectin and insulin concentrations in animals. One study found that glucosamine supplementation induced insulin resistance in animals^37^. A narrative review cited milk proteins to potentially improve postprandial glucose, but more work is needed into the effects of isolated milk proteins (whey, casein), rather than within the dairy matrix, in order for conclusions to be made^38^. Caffeine supplementation did not significantly affect platelet aggregation of ATP signaling in animal studies. A crossover trial investigated the effects of caffeine on post-exercise glucose concentrations in individuals with T1D, and found that it may contribute to late-onset hypoglycemia and should be used with caution^39^.

## Discussion

This scoping review utilized the ODS Researcher Database and a novel web-scraping program to summarize existing evidence supporting dietary supplement use for prevention and treatment of diabetes mellitus. While there were several supplement ingredients that had a larger volume of studies suggesting support of their use (e.g. dietary fiber, selenium, and zinc), the overall results were modest with few human RCTs or meta-analyses (Table 2). In general, we found that most, but not all, ingredients that are currently included in supplements for diabetes had very little to no evidence supporting their use.

Ingredients that had zero articles meeting our inclusion criteria were phosphorus, pantothenic acid, calcium, magnesium, glutamine, isoleucine, tyrosine, choline, and creatine monohydrate. These ingredients are present in a total of 1763 supplements in the ODS database that make a health claim related to diabetes, despite limited evidence. The ingredients with the greatest scientific evidence, fiber, selenium and zinc, totaled 572 products in the ODS database. It is evident that there is a need for greater cohesion between scientific evidence and consumption of dietary supplements.

Many studies were excluded for relying on self-reported diet or supplement intake and associations with reductions in diabetes-related secondary symptoms or for administering treatment as co-supplementation^17^. We and other teams have shown the unreliability of self-reported dietary intake, and its use can lead to the publishing of inaccurate diet-disease relationships^40,41^. In co-supplementation, it is impossible to isolate individual effects of one ingredient if it not examined separately. One common co-supplementation was Vitamin C and Vitamin E, which have been examined in a meta-analysis for their effects on HOMA-IR but concluded no benefit. In the case of reporting a reduction of secondary symptoms of diabetes, such as improved glycemic control, we are unsure if this was the primary goal of the study. It is unclear why these secondary symptoms would be reported without reporting changes in standard measures for presence of diabetes, such as insulin levels, fasting glucose, and HbA1c. Clearly stated research questions a statistical statement of the null and alternative hypothesis and registration with clincialtrials.gov will eliminate these doubts^42^.

Previous reviews on supplement use for diabetes mellitus have concluded mixed results. Twenty-seven meta-analyses were identified in the current study, assessing eight different ingredients. Supplementation of vitamin B6, folate, vitamin C, vitamin E, chromium, and selenium was found to have mixed or null effects on diabetes-related outcomes in meta-analyses. Zinc and fiber were the only two ingredients with consistent positive results in meta-analyses.

There were many notable narrative reviews assessed in the present study, which largely concluded a potential benefit for a particular supplement yet acknowledged the lack of clinical evidence to make such claims. We suspect that the large volume of literature available in the field is not conducive to standard systematic reviews.

Despite lack of clinical evidence, consumers will continue to take dietary supplements for perceived benefit regarding diabetes, thus it is important for healthcare providers to be knowledgeable about common supplements and their potential effects. The role of supplement use for diabetes management, and its potential interactions with other medical treatment approaches, have been reviewed from a pharmacy standpoint and from that of complementary and alternative medicine^11^. As supplement use continues to grow in the US, it is important for healthcare professionals to understand the evidence behind supplements and their potential role as part of medical care. Current supplement use in the US is around 52% of all adults, but use increases with age and is more common among women than men^43^. Among individuals with diabetes, the prevalence is as high as 59%, however this report is from the 2014 NHANES cohort, and the current prevalence may be higher^44^. The most commonly used supplements in this population were lycopene, vitamin D, and vitamin B12.

This study had several strengths. The use of R and the web scrape allowed for thousands of studies indexed in PubMed to be searched based on inclusion of specific keywords. This approach also decreases the potential for human error as it relies on computer extraction of relevant studies rather than manual. Using this method also allows for a rigorous treatment of the which literature to include by the applying the capacity to automatically scrape abstracts. Another strength of this study design is the broad inclusion criteria. As many included studies were conducted in animal models, we were able to assess the effects of supplementation on diabetes-related outcomes in a preclinical model. This is important, as results from animal models can still be used as background to support a dietary supplement claim in conjunction with results from human studies^45^. Finally, exclusion criteria involved removing cross-sectional studies or those relying on self-reported dietary or supplement intake. Self-report dietary intake has been evidenced to be unreliable due to reasons such as recall bias, misestimation of portion sizes, and social desirability bias. To best infer causality between supplement intake and diabetes-related health outcomes, the decision was made to only include controlled experimental trials.

This study is also not without limitations. Included supplements were limited to those indexed in the ODS DSLD. This resource is updated regularly and thoroughly by the ODS and the National Library of Medicine, but it is still possible that there may be relevant supplements that were not found in the search strategy. Additionally, terms related to diabetes (i.e., glycemic control, glucose, insulin, blood sugar, etc.) or diabetes comorbidities were not searched. The purpose of this review was to scope the evidence of current products on the market for diabetes, and not systematically review all supplements related to glycemic control and insulin sensitivity. The effects of individual supplements and diabetes-related outcomes have been systematically reviewed and meta-analyzed previously, including chromium^46,47^, magnesium^48–50^, vitamin D^51^, and vitamin E^52^. Finally, the search for articles was limited to those indexed in PubMed. This allowed our search to be limited to peer-reviewed articles that are pertinent to biomedical sciences and could be searched for pertinent keywords in the title and abstract. However, the authors acknowledge that there may have been potentially relevant studies that were not indexed in PubMed.

In conclusion, there does not exist strong evidence to support the use of many commercial supplements for management of diabetes or its comorbidities. Even existing support is limited due to poor study design and uncontrolled study methods. Before recommendations for supplement use to treat diabetes can be made, there is a need for well-designed human clinical trials to evaluate the role of these ingredients in diabetes-related outcomes.

## Supplementary information

Supplemental Infomation 2

Supplemental Information 1
