# Supplementary material for: Use and abuse of dietary supplements in persons with diabetes
Source: Nutr Diabetes. 2020 Apr 27;10:14. doi: 10.1038/s41387-020-0117-6 (PMC7186221; doi:10.1038/s41387-020-0117-6)
Supplement: Supplementary file 1 — Supplemental Infomation 2 [file 41387_2020_117_MOESM1_ESM.docx]

**Supplementary Information 2**

References for Included Studies

**Water Soluble Vitamins**

*Niacin*

Abdullah, K., Alam, M., Iqbal, Z., & Naseem, I. (2018). Therapeutic effect of vitamin B3 on hyperglycemia, oxidative stress and DNA damage in alloxan induced diabetic rat model. *Biomedicine & Pharmacotherapy*, *105*, 1223-1231. doi: 10.1016/j.biopha.2018.06.085

Li, D., Tian, Y., Guo, J., Sun, W., Lun, Y., & Guo, M. et al. (2013). Nicotinamide supplementation induces detrimental metabolic and epigenetic changes in developing rats. *British Journal Of Nutrition*, *110*(12), 2156-2164. doi: 10.1017/s0007114513001815

Verdoia, M., Schaffer, A., Suryapranata, H., & De Luca, G. (2015). Effects of HDL-modifiers on cardiovascular outcomes: A meta-analysis of randomized trials. *Nutrition, Metabolism And Cardiovascular Diseases*, *25*(1), 9-23. doi: 10.1016/j.numecd.2014.09.003

*Vitamin B6*

Chiazza, F., Cento, A., Collotta, D., Nigro, D., Rosa, G., & Baratta, F. et al. (2017). Protective Effects of Pyridoxamine Supplementation in the Early Stages of Diet-Induced Kidney Dysfunction. *Biomed Research International*, *2017*, 1-12. doi: 10.1155/2017/2682861

Pereira, A., Fernandes, R., Crisóstomo, J., Seiça, R., & Sena, C. (2017). The Sulforaphane and pyridoxamine supplementation normalize endothelial dysfunction associated with type 2 diabetes. *Scientific Reports*, *7*(1). doi: 10.1038/s41598-017-14733-x

Thi Viet Do, Toda, Saibara, & Yagi. (2012). Abnormality in Expression Levels of Gluconeogenesis-Related Genes by High-Dose Supplementation with Pyridoxamine in Mice. *International Journal For Vitamin And Nutrition Research*, *82*(1), 34-40. doi: 10.1024/0300-9831/a000092

Yan, M., & Khalil, H. (2017). Vitamin supplements in type 2 diabetes mellitus management: A review. *Diabetes & Metabolic Syndrome: Clinical Research & Reviews*, *11*, S589-S595. doi: 10.1016/j.dsx.2017.04.009

*Biotin*

McCarty, M. (2000). Toward a wholly nutritional therapy for type 2 diabetes. Medical Hypotheses, 54(3), 483-487. doi: 10.1054/mehy.1999.0881

McCarty, M. (2005). Nutraceutical resources for diabetes prevention – an update. Medical Hypotheses, 64(1), 151-158. doi: 10.1016/j.mehy.2004.03.036

Turgut, M., Cinar, V., Pala, R., Tuzcu, M., Orhan, C., & Telceken, H. et al. (2018). Biotin and chromium histidinate improve glucose metabolism and proteins expression levels of IRS-1, PPAR-γ, and NF-κB in exercise-trained rats. Journal Of The International Society Of Sports Nutrition, 15(1). doi: 10.1186/s12970-018-0249-4

Valdes-Ramos, R., Guadarrama-Lopez, A., Martinez-Carrillo, B., & Benitez-Arciniega, A. (2015). Vitamins and type 2 diabetes mellitus. Endocrine, Metabolic & Immune Disorders, 15(1). doi: 10.2174/1871530314666141111103217

*Folic Acid*

Akbari, M., Tabrizi, R., Lankarani, K., Heydari, S., Karamali, M., & Kashanian, M. et al. (2018). The Effects of Folate Supplementation on Diabetes Biomarkers Among Patients with Metabolic Diseases: A Systematic Review and Meta-Analysis of Randomized Controlled Trials. *Hormone And Metabolic Research*, *50*(02), 93-105. doi: 10.1055/s-0043-125148

Alian, Z., Hashemipour, M., Dehkordi, E., Hovsepian, S., Amini, M., Moadab, M., & Javanmard, S. (2012). The Effects of Folic Acid on Markers of Endothelial Function in Patients with Type 1 Diabetes Mellitus. *Medical Archives*, *66*(1), 12. doi: 10.5455/medarh.2012.66.12-15

Sudchada, P., Saokaew, S., Sridetch, S., Incampa, S., Jaiyen, S., & Khaithong, W. (2012). Effect of folic acid supplementation on plasma total homocysteine levels and glycemic control in patients with type 2 diabetes: A systematic review and meta-analysis. *Diabetes Research And Clinical Practice*, *98*(1), 151-158. doi: 10.1016/j.diabres.2012.05.027

Talari, H., Rafiee, M., Farrokhian, A., Raygan, F., Bahmani, F., & Darooghegi Mofrad, M. et al. (2016). The Effects of Folate Supplementation on Carotid Intima-Media Thickness and Metabolic Status in Patients with Metabolic Syndrome. *Annals Of Nutrition And Metabolism*, *69*(1), 41-50. doi: 10.1159/000448295

Xu, R., Kong, X., Xu, B., Song, Y., Ji, M., & Zhao, M. et al. (2017). Longitudinal association between fasting blood glucose concentrations and first stroke in hypertensive adults in China: effect of folic acid intervention. *The American Journal Of Clinical Nutrition*, *105*(3), 564-570. doi: 10.3945/ajcn.116.145656

Zhao, J., Schooling, C., & Zhao, J. (2018). The effects of folate supplementation on glucose metabolism and risk of type 2 diabetes: a systematic review and meta-analysis of randomized controlled trials. *Annals Of Epidemiology*, *28*(4), 249-257.e1. doi: 10.1016/j.annepidem.2018.02.001

*Vitamin B12*

Jayabalan, B., & Low, L. (2016). Vitamin B supplementation for diabetic peripheral neuropathy. *Singapore Medical Journal*, *57*(02), 55-59. doi: 10.11622/smedj.2016027

Kwok, T., Lee, J., Ma, R., Wong, S., Kung, K., & Lam, A. et al. (2017). A randomized placebo controlled trial of vitamin B 12 supplementation to prevent cognitive decline in older diabetic people with borderline low serum vitamin B 12. *Clinical Nutrition*, *36*(6), 1509-1515. doi: 10.1016/j.clnu.2016.10.018

Lin, H., Chung, C., Chang, C., Wang, M., Lin, J., & Shen, M. (2007). Hyperhomocysteinemia, Deep Vein Thrombosis and Vitamin B12 Deficiency in a Metformin-treated Diabetic Patient. *Journal Of The Formosan Medical Association*, *106*(9), 774-778. doi: 10.1016/s0929-6646(08)60039-x

Valdes-Ramos, R., Guadarrama-Lopez, A., Martinez-Carrillo, B., & Benitez-Arciniega, A. (2015). Vitamins and type 2 diabetes mellitus. Endocrine, Metabolic & Immune Disorders, 15(1). doi: 10.2174/1871530314666141111103217

Yan, M., & Khalil, H. (2017). Vitamin supplements in type 2 diabetes mellitus management: A review. *Diabetes & Metabolic Syndrome: Clinical Research & Reviews*, *11*, S589-S595. doi: 10.1016/j.dsx.2017.04.009

*Vitamin C*

Aluwong, T., Ayo, J., Kpukple, A., & Oladipo, O. (2016). Amelioration of Hyperglycaemia, Oxidative Stress and Dyslipidaemia in Alloxan-Induced Diabetic Wistar Rats Treated with Probiotic and Vitamin C. *Nutrients*, *8*(5), 151. doi: 10.3390/nu8050151

Ashor, A., Werner, A., Lara, J., Willis, N., Mathers, J., & Siervo, M. (2017). Effects of vitamin C supplementation on glycaemic control: a systematic review and meta-analysis of randomised controlled trials. *European Journal Of Clinical Nutrition*, *71*(12), 1371-1380. doi: 10.1038/ejcn.2017.24

Badr, G., Bashandy, S., Ebaid, H., Mohany, M., & Sayed, D. (2011). Vitamin C supplementation reconstitutes polyfunctional T cells in streptozotocin-induced diabetic rats. *European Journal Of Nutrition*, *51*(5), 623-633. doi: 10.1007/s00394-011-0176-5

Bartlett, H., & Eperjesi, F. (2008). Nutritional supplementation for type 2 diabetes: a systematic review. *Ophthalmic And Physiological Optics*, *28*(6), 503-523. doi: 10.1111/j.1475-1313.2008.00595.x

Bolignano, D., Cernaro, V., Gembillo, G., Baggetta, R., Buemi, M., & D’Arrigo, G. (2017). Antioxidant agents for delaying diabetic kidney disease progression: A systematic review and meta-analysis. *PLOS ONE*, *12*(6), e0178699. doi: 10.1371/journal.pone.0178699

de Paula, T., Kramer, C., Viana, L., & Azevedo, M. (2017). Effects of individual micronutrients on blood pressure in patients with type 2 diabetes: a systematic review and meta-analysis of randomized clinical trials. *Scientific Reports*, *7*(1). doi: 10.1038/srep40751

El-Aal, A., El-Ghffar, E., Ghali, A., Zughbur, M., & Sirdah, M. (2018). The effect of vitamin C and/or E supplementations on type 2 diabetic adult males under metformin treatment: A single-blinded randomized controlled clinical trial. *Diabetes & Metabolic Syndrome: Clinical Research & Reviews*, *12*(4), 483-489. doi: 10.1016/j.dsx.2018.03.013

Greń, A. (2013). Effects of Vitamin E, C and D Supplementation on Inflammation and Oxidative Stress in Streptozotocin-Induced Diabetic Mice. *International Journal For Vitamin And Nutrition Research*, *83*(3), 168-175. doi: 10.1024/0300-9831/a000156

Grzebyk, E., & Piwowar, A. (2016). Inhibitory actions of selected natural substances on formation of advanced glycation endproducts and advanced oxidation protein products. *BMC Complementary And Alternative Medicine*, *16*(1). doi: 10.1186/s12906-016-1353-0

Hejazi, N., Mazloom, Z., & Ekramzadeh, M. (2013). Efficacy of Supplementary Vitamins C and E on Anxiety, Depression and Stress in Type 2 Diabetic Patients: A Randomized, Single-blind, Placebo-controlled Trial. *Pakistan Journal Of Biological Sciences*, *16*(22), 1597-1600. doi: 10.3923/pjbs.2013.1597.1600

Hejazi, N., Mazloom, Z., Dabbaghman, M., Tabatabaei, H., Ahmadi, A., & Ansar, H. (2011). Effect of Vitamin C Supplementation on Postprandial Oxidative Stress and Lipid Profile in Type 2 Diabetic Patients. *Pakistan Journal Of Biological Sciences*, *14*(19), 900-904. doi: 10.3923/pjbs.2011.900.904

Khodaeian, M., Tabatabaei-Malazy, O., Qorbani, M., Farzadfar, F., Amini, P., & Larijani, B. (2015). Effect of vitamins C and E on insulin resistance in diabetes: a meta-analysis study. *European Journal Of Clinical Investigation*, *45*(11), 1161-1174. doi: 10.1111/eci.12534

Owu, D., Nwokocha, C., Ikpi, D., & Ogar, E. (2016). Effect of Vitamin C Supplementation on Platelet Aggregation and Serum Electrolytes Levels in Streptozotocin-Induced Diabetes Mellitus in Rats. *Nigerian Journal Of Physiological Science*, *31*(1), 55-61.

Song, Y., Cook, N., Albert, C., Van Denburgh, M., & Manson, J. (2009). Effects of vitamins C and E and β-carotene on the risk of type 2 diabetes in women at high risk of cardiovascular disease: a randomized controlled trial. *The American Journal Of Clinical Nutrition*, *90*(2), 429-437. doi: 10.3945/ajcn.2009.27491

Tabatabaei-Malazy, O., Nikfar, S., Larijani, B., & Abdollahi, M. (2015). Influence of Ascorbic Acid Supplementation on Type 2 Diabetes Mellitus in Observational and Randomized Controlled Trials; A Systematic Review with Meta-Analysis. *Journal Of Pharmacy & Pharmaceutical Sciences*, *17*(4), 554. doi: 10.18433/j3zg6r

**Fat-soluble Vitamins**

*Vitamin E*

Aghadavod, E., Soleimani, A., Hamidi, G., Keneshlou, F., Heidari, A., & Asemi, Z. (2018). Effects of High-dose Vitamin E Supplementation on Markers of Cardiometabolic Risk and Oxidative Stress in Patients with Diabetic Nephropathy: a Randomized Double-blinded Controlled Trial. *Iranian Journal of Kidney Diseases, 12*(3), 156-162.

de Oliveira, A. M., Rondó, P. H. C., Luzia, L. A., D’Abronzo, F. H., & Illison, V. K. (2011). The effects of lipoic acid and α-tocopherol supplementation on the lipid profile and insulin sensitivity of patients with type 2 diabetes mellitus: a randomized, double-blind, placebo-controlled trial. *Diabetes research and clinical practice*, *92*(2), 253-260.

El-Aal, A., El-Ghffar, E., Ghali, A., Zughbur, M., & Sirdah, M. (2018). The effect of vitamin C and/or E supplementations on type 2 diabetic adult males under metformin treatment: A single-blinded randomized controlled clinical trial. *Diabetes & Metabolic Syndrome: Clinical Research & Reviews*, *12*(4), 483-489. doi: 10.1016/j.dsx.2018.03.013

Fang, F., Kang, Z., & Wong, C. (2010). Vitamin E tocotrienols improve insulin sensitivity through activating peroxisome proliferator‐activated receptors. *Molecular nutrition & food research*, *54*(3), 345-352.

Greń, A. (2013). Effects of Vitamin E, C and D Supplementation on Inflammation and Oxidative Stress in Streptozotocin-Induced Diabetic Mice. *International Journal for Vitamin and Nutrition Research*, *83*(3), 168-175. doi: 10.1024/0300-9831/a000156

Gupta, S., Sharma, T. K., Kaushik, G. G., & Shekhawat, V. P. (2011). Vitamin E supplementation may ameliorate oxidative stress in type 1 diabetes mellitus patients. *Clinical laboratory*, *57*(5-6), 379-386.

Hani, H., Allaudin, Z., Mohd-Lila, M., Sarsaifi, K., Rasouli, M., & Tam, Y. et al. (2017). Improvement of isolated caprine islet survival and functionality in vitro by enhancing of PDX1 gene expression. *Xenotransplantation*, *24*(3), e12302. doi: 10.1111/xen.12302

Khatami, P., Soleimani, A., Sharifi, N., Aghadavod, E., & Asemi, Z. (2016). The effects of high-dose vitamin E supplementation on biomarkers of kidney injury, inflammation, and oxidative stress in patients with diabetic nephropathy: A randomized, double-blind, placebo-controlled trial. *Journal Of Clinical Lipidology*, *10*(4), 922-929. doi: 10.1016/j.jacl.2016.02.021

Khodaeian, M., Tabatabaei-Malazy, O., Qorbani, M., Farzadfar, F., Amini, P., & Larijani, B. (2015). Effect of vitamins C and E on insulin resistance in diabetes: a meta-analysis study. *European Journal Of Clinical Investigation*, *45*(11), 1161-1174. doi: 10.1111/eci.12534

Magielse, J., Verlaet, A., Breynaert, A., Keenoy, B. M. Y., Apers, S., Pieters, L., & Hermans, N. (2014). Investigation of the in vivo antioxidative activity of C ynara scolymus (artichoke) leaf extract in the streptozotocin‐induced diabetic rat. *Molecular nutrition & food research*, *58*(1), 211-215.

Maier, H. M., Ilich, J. Z., Kim, J. S., & Spicer, M. T. (2013). Nutrition supplementation for diabetic wound healing: a systematic review of current literature. *Skinmed*, *11*(4), 217-24.

Manning, P. J., Sutherland, W. H. F., Williams, S. M., Walker, R. J., Berry, E. A., De Jong, S. A., & Ryalls, A. R. (2013). The effect of lipoic acid and vitamin E therapies in individuals with the metabolic syndrome. *Nutrition, Metabolism and Cardiovascular Diseases*, *23*(6), 543-549.

Mazloom, Z., Ekramzadeh, M., & Hejazi, N. (2013). Efﬁcacy of Supplementary Vitamins C and E on Anxiety, Depression and Stress in Type 2 Diabetic Patients: A Randomized, Single-blind, Placebo-controlled Trial. *Pakistan Journal of Biological Sciences*, *16*(22), 1597-1600.

Montero, D., Walther, G., Stehouwer, C. D. A., Houben, A. J. H. M., Beckman, J. A., & Vinet, A. (2014). Effect of antioxidant vitamin supplementation on endothelial function in type 2 diabetes mellitus: a systematic review and meta‐analysis of randomized controlled trials. *Obesity reviews*, *15*(2), 107-116.

Rafighi, Z., Shiva, A., Arab, S., & Yusuf, R. M. (2013). Association of dietary vitamin C and E intake and antioxidant enzymes in type 2 diabetes mellitus patients. *Global journal of health science*, *5*(3), 183.

Rashid, S. (2014). Effect of alphatocopherol on diameter of proximal convoluted tubules of kidney in diabetic mice. *J Pak Med Assoc*, *64*, 49-52.

Roldi, L., Pereira, R., Tronchini, E., Rizo, G., Scoaris, C., Zanoni, J., & Natali, M. (2009). Vitamin E (α-tocopherol) supplementation in diabetic rats: effects on the proximal colon. *BMC Gastroenterology*, *9*(1). doi: 10.1186/1471-230x-9-88

Shin, J., Yang, S., & Lim, Y. (2016). Gamma-tocopherol supplementation ameliorated hyper-inflammatory response during the early cutaneous wound healing in alloxan-induced diabetic mice. *Experimental Biology and Medicine*, *242*(5), 505-515. doi: 10.1177/1535370216683836

Stonehouse, W., Brinkworth, G., Thompson, C., & Abeywardena, M. (2016). Short term effects of palm-tocotrienol and palm-carotenes on vascular function and cardiovascular disease risk: A randomised controlled trial. *Atherosclerosis*, *254*, 205-214. doi: 10.1016/j.atherosclerosis.2016.10.027

Suksomboon, N., Poolsup, N., & Sinprasert, S. (2011). Effects of vitamin E supplementation on glycaemic control in type 2 diabetes: systematic review of randomized controlled trials. *Journal of clinical pharmacy and therapeutics*, *36*(1), 53-63.

Weng-Yew, W., & Brown, L. (2011). Nutrapharmacology of tocotrienols for metabolic syndrome. *Current pharmaceutical design*, *17*(21), 2206-2214.

Xu, R., Zhang, S., Tao, A., Chen, G., & Zhang, M. (2014). Influence of Vitamin E Supplementation on Glycaemic Control: A Meta-Analysis of Randomised Controlled Trials. *Plos ONE*, *9*(4), e95008. doi: 10.1371/journal.pone.0095008

**Minerals**

*Chromium*

Abdourahman, A., & Edwards, J. G. (2008). Chromium supplementation improves glucose tolerance in diabetic Goto‐Kakizaki rats. *IUBMB life*, *60*(8), 541-548.

Abdollahi, M., Farshchi, A., Nikfar, S., & Seyedifar, M. (2013). Effect of chromium on glucose and lipid profiles in patients with type 2 diabetes; a meta-analysis review of randomized trials. *Journal of Pharmacy & Pharmaceutical Sciences*, *16*(1), 99-114.

Anderson, R. A. (2008). Chromium and polyphenols from cinnamon improve insulin sensitivity: plenary lecture. *Proceedings of the Nutrition Society*, *67*(1), 48-53.

Bailey, C. H. (2014). Improved meta-analytic methods show no effect of chromium supplements on fasting glucose. *Biological trace element research*, *157*(1), 1-8.

Bartlett, H. E., & Eperjesi, F. (2008). Nutritional supplementation for type 2 diabetes: a systematic review. *Ophthalmic and Physiological Optics*, *28*(6), 503-523.

Cefalu, W. T., Rood, J., Pinsonat, P., Qin, J., Sereda, O., Levitan, L., & Wang, Z. Q. (2010). Characterization of the metabolic and physiologic response to chromium supplementation in subjects with type 2 diabetes mellitus. *Metabolism*, *59*(5), 755-762.

Costello, R. B., Dwyer, J. T., & Bailey, R. L. (2016). Chromium supplements for glycemic control in type 2 diabetes: limited evidence of effectiveness. *Nutrition reviews*, *74*(7), 455-468.

Feng, W., Wu, H., Li, Q., Zhou, Z., Chen, Y., Zhao, T., & Wu, X. (2015). Evaluation of 90-day repeated dose oral toxicity, glycometabolism, learning and memory ability, and related enzyme of chromium malate supplementation in sprague-dawley rats. *Biological trace element research*, *168*(1), 181-195.

Feng, W., Zhao, T., Mao, G., Wang, W., Feng, Y., Li, F., & Wu, X. (2015). Type 2 diabetic rats on diet supplemented with chromium malate show improved glycometabolism, glycometabolism-related enzyme levels and lipid metabolism. *PLoS One*, *10*(5), e0125952.

Guimaraes, M. M., Carvalho, A. C. M. S., & Silva, M. S. (2016). Effect of chromium supplementation on the glucose homeostasis and anthropometry of type 2 diabetic patients: Double blind, randomized clinical trial: Chromium, glucose homeostasis and anthropometry. *Journal of Trace Elements in Medicine and Biology*, *36*, 65-72.

Guimarães, M. M., Martins Silva Carvalho, A. C., & Silva, M. S. (2013). Chromium nicotinate has no effect on insulin sensitivity, glycemic control, and lipid profile in subjects with type 2 diabetes. *Journal of the American College of Nutrition*, *32*(4), 243-250.

Hoffman, N. J., Penque, B. A., Habegger, K. M., Sealls, W., Tackett, L., & Elmendorf, J. S. (2014). Chromium enhances insulin responsiveness via AMPK. *The Journal of nutritional biochemistry*, *25*(5), 565-572.

Huang, H., Chen, G., Dong, Y., Zhu, Y., & Chen, H. (2018). Chromium supplementation for adjuvant treatment of type 2 diabetes mellitus: results from a pooled analysis. *Molecular nutrition & food research*, *62*(1), 1700438.

Jain, S. K., Croad, J. L., Velusamy, T., Rains, J. L., & Bull, R. (2010). Chromium dinicocysteinate supplementation can lower blood glucose, CRP, MCP‐1, ICAM‐1, creatinine, apparently mediated by elevated blood vitamin C and adiponectin and inhibition of NFκB, Akt, and Glut‐2 in livers of zucker diabetic fatty rats. *Molecular nutrition & food research*, *54*(9), 1371-1380.

Jain, S. K., Kahlon, G., Morehead, L., Dhawan, R., Lieblong, B., Stapleton, T., & Bass III, P. F. (2012). Effect of chromium dinicocysteinate supplementation on circulating levels of insulin, TNF‐α, oxidative stress, and insulin resistance in type 2 diabetic subjects: Randomized, double‐blind, placebo‐controlled study. *Molecular nutrition & food research*, *56*(8), 1333-1341.

Kolahian, S., Sadri, H., Shahbazfar, A. A., Amani, M., Mazadeh, A., & Mirani, M. (2015). The effects of leucine, zinc, and chromium supplements on inflammatory events of the respiratory system in type 2 diabetic rats. *PloS one*, *10*(7), e0133374.

Komorowski, J. R., Tuzcu, M., Sahin, N., Juturu, V., Orhan, C., Ulas, M., & Sahin, K. (2012). Chromium picolinate modulates serotonergic properties and carbohydrate metabolism in a rat model of diabetes. *Biological trace element research*, *149*(1), 50-56.

Król, E., Krejpcio, Z., & Iwanik, K. (2014). Supplementary chromium (III) propionate complex does not protect against insulin resistance in high-fat-fed rats. *Biological trace element research*, *157*(2), 147-155.

Król, E., Krejpcio, Z., Michalak, S., Wójciak, R. W., & Bogdański, P. (2012). Effects of combined dietary chromium (III) propionate complex and thiamine supplementation on insulin sensitivity, blood biochemical indices, and mineral levels in high-fructose-fed rats. *Biological trace element research*, *150*(1-3), 350-359.

Li, F., Wu, X., Zhao, T., Zhang, M., Zhao, J., Mao, G., & Yang, L. (2011). Anti-diabetic properties of chromium citrate complex in alloxan-induced diabetic rats. *Journal of Trace Elements in Medicine and Biology*, *25*(4), 218-224.

Li, F., Wu, X., Zou, Y., Zhao, T., Zhang, M., Feng, W., & Yang, L. (2012). Comparing anti-hyperglycemic activity and acute oral toxicity of three different trivalent chromium complexes in mice. *Food and chemical toxicology*, *50*(5), 1623-1631.

Liu, L., Jin, W., & Lv, J. P. (2010). Oral administration of the high-chromium yeast improve blood plasma variables and pancreatic islet tissue in diabetic mice. *Biological trace element research*, *138*(1-3), 250-264.

Masharani, U., Gjerde, C., McCoy, S., Maddux, B. A., Hessler, D., Goldfine, I. D., & Youngren, J. F. (2012). Chromium supplementation in non-obese non-diabetic subjects is associated with a decline in insulin sensitivity. *BMC endocrine disorders*, *12*(1), 31.

Paiva, A. N., de Lima, J. G., de Medeiros, A. C., Figueiredo, H. A., de Andrade, R. L., Ururahy, M. A., ... & Almeida, M. D. G. (2015). Beneficial effects of oral chromium picolinate supplementation on glycemic control in patients with type 2 diabetes: a randomized clinical study. *Journal of Trace Elements in Medicine and Biology*, *32*, 66-72.

Refaie, F. M., Esmat, A. Y., Mohamed, A. F., & Nour, W. H. A. (2009). Effect of chromium supplementation on the diabetes induced-oxidative stress in liver and brain of adult rats. *Biometals*, *22*(6), 1075.

Sadri, H., Larki, N. N., & Kolahian, S. (2017). Hypoglycemic and hypolipidemic effects of leucine, zinc, and chromium, alone and in combination, in rats with type 2 diabetes. *Biological trace element research*, *180*(2), 246-254.

Sahin, K., Tuzcu, M., Orhan, C., Gencoglu, H., Ulas, M., Atalay, M., & Komorowski, J. R. (2012). The effects of chromium picolinate and chromium histidinate administration on NF-κB and Nrf2/HO-1 pathway in the brain of diabetic rats. *Biological trace element research*, *150*(1-3), 291-296.

Sahin, K., Tuzcu, M., Orhan, C., Sahin, N., Kucuk, O., Ozercan, I. H., & Komorowski, J. R. (2013). Anti-diabetic activity of chromium picolinate and biotin in rats with type 2 diabetes induced by high-fat diet and streptozotocin. *British Journal of Nutrition*, *110*(2), 197-205.

Sreejayan, N., Dong, F., Kandadi, M. R., Yang, X., & Ren, J. (2008). Chromium alleviates glucose intolerance, insulin resistance, and hepatic ER stress in obese mice. *Obesity*, *16*(6), 1331-1337.

Staniek, H., Krejpcio, Z., & Wieczorek, D. (2016). The effects of high dietary doses of chromium (III) complex with propionic acid on nutritional and selected blood indices in healthy female rats. *Biological trace element research*, *171*(1), 192-200.

Suksomboon, N., Poolsup, N., & Yuwanakorn, A. (2014). Systematic review and meta‐analysis of the efficacy and safety of chromium supplementation in diabetes. *Journal of clinical pharmacy and therapeutics*, *39*(3), 292-306.

Ulas, M., Orhan, C., Tuzcu, M., Ozercan, I. H., Sahin, N., Gencoglu, H., & Sahin, K. (2015). Anti-diabetic potential of chromium histidinate in diabetic retinopathy rats. *BMC complementary and alternative medicine*, *15*(1), 16.

Yeghiazaryan, K., H Schild, H., & Golubnitschaja, O. (2012). Chromium-picolinate therapy in diabetes care: individual outcomes require new guidelines and navigation by predictive diagnostics. *Infectious Disorders-Drug Targets (Formerly Current Drug Targets-Infectious Disorders)*, *12*(5), 332-339.

Yeghiazaryan, K., Peeva, V., Shenoy, A., H Schild, H., & Golubnitschaja, O. (2011). Chromium-picolinate therapy in diabetes care: molecular and subcellular profiling revealed a necessity for individual outcome prediction, personalised treatment algorithms & new guidelines. *Infectious Disorders-Drug Targets (Formerly Current Drug Targets-Infectious Disorders)*, *11*(2), 188-195.

Yilmaz, Z., Piracha, F., Anderson, L., & Mazzola, N. (2017). Supplements for diabetes mellitus: A review of the literature. *Journal of pharmacy practice*, *30*(6), 631-638.

Yin, R. V., & Phung, O. J. (2015). Effect of chromium supplementation on glycated hemoglobin and fasting plasma glucose in patients with diabetes mellitus. *Nutrition journal*, *14*(1), 14.

*Potassium*

Asghar, M., Monjok, E., Kouamou, G., Ohia, S. E., Bagchi, D., & Lokhandwala, M. F. (2007). Super CitriMax (HCA-SX) attenuates increases in oxidative stress, inflammation, insulin resistance, and body weight in developing obese Zucker rats. *Molecular and cellular biochemistry*, *304*(1-2), 93.

Chatterjee, R., Slentz, C., Davenport, C. A., Johnson, J., Lin, P. H., Muehlbauer, M., & Edelman, D. (2017). Effects of potassium supplements on glucose metabolism in African Americans with prediabetes: a pilot trial. *The American Journal of Clinical Nutrition*, *106*(6), 1431-1438.

Martini, L. A., Catania, A. S., & Ferreira, S. R. (2010). Role of vitamins and minerals in prevention and management of type 2 diabetes mellitus. *Nutrition reviews*, *68*(6), 341-354.

*Selenium*

Agbor, G. A., Vinson, J. A., Patel, S., Patel, K., Scarpati, J., Shiner, D., & Tompkins, T. A. (2007). Effect of selenium-and glutathione-enriched yeast supplementation on a combined atherosclerosis and diabetes hamster model. *Journal of agricultural and food chemistry*, *55*(21), 8731-8736.

Asemi, Z., Jamilian, M., Mesdaghinia, E., & Esmaillzadeh, A. (2015). Effects of selenium supplementation on glucose homeostasis, inflammation, and oxidative stress in gestational diabetes: Randomized, double-blind, placebo-controlled trial. *Nutrition*, *31*(10), 1235-1242.

Bahmani, F., Kia, M., Soleimani, A., Asemi, Z., & Esmaillzadeh, A. (2016). Effect of selenium supplementation on glycemic control and lipid profiles in patients with diabetic nephropathy. *Biological trace element research*, *172*(2), 282-289.

Bahmani, F., Kia, M., Soleimani, A., Mohammadi, A. A., & Asemi, Z. (2016). The effects of selenium supplementation on biomarkers of inflammation and oxidative stress in patients with diabetic nephropathy: a randomised, double-blind, placebo-controlled trial. *British Journal of Nutrition*, *116*(7), 1222-1228.

Chen, H., Qiu, Q., Zou, C., Dou, L., & Liang, J. (2015). Regulation of hepatic carbohydrate metabolism by selenium during diabetes. *Chemico-biological interactions*, *232*, 1-6.

Faghihi, T., Radfar, M., Barmal, M., Amini, P., Qorbani, M., Abdollahi, M., & Larijani, B. (2014). A randomized, placebo-controlled trial of selenium supplementation in patients with type 2 diabetes: effects on glucose homeostasis, oxidative stress, and lipid profile. *American journal of therapeutics*, *21*(6), 491-495.

Farrokhian, A., Bahmani, F., Taghizadeh, M., Mirhashemi, S. M., Aarabi, M. H., Raygan, F., & Asemi, Z. (2016). Selenium supplementation affects insulin resistance and serum hs-CRP in patients with type 2 diabetes and coronary heart disease. *Hormone and metabolic research*, *48*(04), 263-268.

Fatmi, W., Kechrid, Z., Nazıroğlu, M., & Flores-Arce, M. (2013). Selenium supplementation modulates zinc levels and antioxidant values in blood and tissues of diabetic rats fed zinc-deficient diet. *Biological trace element research*, *152*(2), 243-250.

Faure, P., Ramon, O., Favier, A., & Halimi, S. (2004). Selenium supplementation decreases nuclear factor‐kappa B activity in peripheral blood mononuclear cells from type 2 diabetic patients. *European journal of clinical investigation*, *34*(7), 475-481.

Febiyanto, N., Yamazaki, C., Kameo, S., Sari, D. K., Puspitasari, I. M., Sunjaya, D. K., & Koyama, H. (2018). Effects of selenium supplementation on the diabetic condition depend on the baseline selenium status in KKAy mice. *Biological trace element research*, *181*(1), 71-81.

Jablonska, E., Reszka, E., Gromadzinska, J., Wieczorek, E., Krol, M., Raimondi, S., & Wasowicz, W. (2016). The effect of selenium supplementation on glucose homeostasis and the expression of genes related to glucose metabolism. *Nutrients*, *8*(12), 772.

Jamilian, M., Samimi, M., Ebrahimi, F. A., Aghadavod, E., Mohammadbeigi, R., Rahimi, M., & Asemi, Z. (2018). Effects of selenium supplementation on gene expression levels of inflammatory cytokines and vascular endothelial growth factor in patients with gestational diabetes. *Biological trace element research*, *181*(2), 199-206.

Janbakhsh, A., Mansouri, F., Vaziri, S., Sayad, B., Afsharian, M., Rahimi, M., & Salari, F. (2013). Effect of selenium on immune response against hepatitis B vaccine with accelerated method in insulin-dependent diabetes mellitus patients. *Caspian journal of internal medicine*, *4*(1), 603.

Kim, J. E., Choi, S. I., Lee, H. R., Hwang, I. S., Lee, Y. J., An, B. S., ... & Hwang, D. Y. (2012). Selenium significantly inhibits adipocyte hypertrophy and abdominal fat accumulation in OLETF rats via induction of fatty acid β-oxidation. *Biological trace element research*, *150*(1-3), 360-370.

Koyama, H., Abdulah, R., Yamazaki, C., & Kameo, S. (2013). Selenium supplementation trials for cancer prevention and the subsequent risk of type 2 diabetes mellitus: selenium and vitamin E cancer prevention trial and after. *Nihon eiseigaku zasshi. Japanese journal of hygiene*, *68*(1), 1-10.

Labunskyy, V. M., Lee, B. C., Handy, D. E., Loscalzo, J., Hatfield, D. L., & Gladyshev, V. N. (2011). Both maximal expression of selenoproteins and selenoprotein deficiency can promote development of type 2 diabetes-like phenotype in mice. *Antioxidants & redox signaling*, *14*(12), 2327-2336.

Mao, S., Zhang, A., & Huang, S. (2014). Selenium supplementation and the risk of type 2 diabetes mellitus: a meta-analysis of randomized controlled trials.

Mueller, A. S., Bosse, A. C., Most, E., Klomann, S. D., Schneider, S., & Pallauf, J. (2009). Regulation of the insulin antagonistic protein tyrosine phosphatase 1B by dietary Se studied in growing rats. *The Journal of nutritional biochemistry*, *20*(4), 235-247.

Ogawa-Wong, A. N., Berry, M. J., & Seale, L. A. (2016). Selenium and metabolic disorders: an emphasis on type 2 diabetes risk. *Nutrients*, *8*(2), 80.

Panchal, S. K., Wanyonyi, S., & Brown, L. (2017). Selenium, vanadium, and chromium as micronutrients to improve metabolic syndrome. *Current hypertension reports*, *19*(3), 10.

Stranges, S., Marshall, J. R., Natarajan, R., Donahue, R. P., Trevisan, M., Combs, G. F., & Reid, M. E. (2007). Effects of long-term selenium supplementation on the incidence of type 2 diabetes: a randomized trial. *Annals of internal medicine*, *147*(4), 217-223.

Tanko, Y., Jimoh, A., Ahmed, A., Adam, A., Ejeh, L., Mohammed, A., & Ayo, J. O. (2016). Effects of selenium yeast on blood glucose and antioxidant biomarkers in cholesterol fed diet induced type 2 diabetes mellitus in wistar rats. *Nigerian Journal of Physiological Sciences*, *31*(2), 147-152.

Vinceti, M., Filippini, T., & Rothman, K. J. (2018). Selenium exposure and the risk of type 2 diabetes: a systematic review and meta-analysis.

Wang, C., Yang, S., Zhang, N., Mu, Y., Ren, H., Wang, Y., & Li, K. (2014). Long-term supranutritional supplementation with selenate decreases hyperglycemia and promotes fatty liver degeneration by inducing hyperinsulinemia in diabetic db/db mice. *PLoS One*, *9*(7), e101315.

Wang, N., Tan, H. Y., Li, S., Xu, Y., Guo, W., & Feng, Y. (2017). Supplementation of micronutrient selenium in metabolic diseases: its role as an antioxidant. *Oxidative medicine and cellular longevity*, *2017*.

*Sodium*

Bjornstad, P., Maahs, D. M., Roncal, C. A., Snell‐Bergeon, J. K., Shah, V. N., Milagres, T., & Garg, S. (2018). Role of bicarbonate supplementation on urine uric acid crystals and diabetic tubulopathy in adults with type 1 diabetes. *Diabetes, Obesity and Metabolism*, *20*(7), 1776-1780.

Carlström, M., Larsen, F. J., Nyström, T., Hezel, M., Borniquel, S., Weitzberg, E., & Lundberg, J. O. (2010). Dietary inorganic nitrate reverses features of metabolic syndrome in endothelial nitric oxide synthase-deficient mice. *Proceedings of the National Academy of Sciences*, *107*(41), 17716-17720.

Gheibi, S., Jeddi, S., Carlström, M., Gholami, H., & Ghasemi, A. (2018). Effects of long-term nitrate supplementation on carbohydrate metabolism, lipid profiles, oxidative stress, and inflammation in male obese type 2 diabetic rats. *Nitric Oxide*, *75*, 27-41.

Matheus, V. A., Monteiro, L. C. S., Oliveira, R. B., Maschio, D. A., & Collares-Buzato, C. B. (2017). Butyrate reduces high-fat diet-induced metabolic alterations, hepatic steatosis and pancreatic beta cell and intestinal barrier dysfunctions in prediabetic mice. *Experimental Biology and Medicine*, *242*(12), 1214-1226.

Miyazaki, T., Shirakami, Y., Kubota, M., Ideta, T., Kochi, T., Sakai, H., & Shimizu, M. (2016). Sodium alginate prevents progression of non-alcoholic steatohepatitis and liver carcinogenesis in obese and diabetic mice. *Oncotarget*, *7*(9), 10448.

Norouzirad, R., Gholami, H., Ghanbari, M., Hedayati, M., González-Muniesa, P., Jeddi, S., & Ghasemi, A. (2019). Dietary inorganic nitrate attenuates hyperoxia-induced oxidative stress in obese type 2 diabetic male rats. *Life sciences*.

Roshanravan, N., Mahdavi, R., Alizadeh, E., Jafarabadi, M. A., Hedayati, M., Ghavami, A., & Ostadrahimi, A. (2017). Effect of butyrate and inulin supplementation on glycemic status, lipid profile and glucagon-like peptide 1 level in patients with type 2 diabetes: A randomized double-blind, placebo-controlled trial. *Hormone and Metabolic Research*, *49*(11), 886-891.

*Zinc*

Aziz, N. M., Kamel, M. Y., Mohamed, M. S., & Ahmed, S. M. (2018). Antioxidant, anti-inflammatory, and anti-apoptotic effects of zinc supplementation in testes of rats with experimentally induced diabetes. *Applied Physiology, Nutrition, and Metabolism*, *43*(10), 1010-1018.

Barman, S., Pradeep, S. R., & Srinivasan, K. (2017). Zinc supplementation mitigates its dyshomeostasis in experimental diabetic rats by regulating the expression of zinc transporters and metallothionein. *Metallomics*, *9*(12), 1765-1777.

Barman, S., & Srinivasan, K. (2017). Attenuation of oxidative stress and cardioprotective effects of zinc supplementation in experimental diabetic rats. *British Journal of Nutrition*, *117*(3), 335-350.

Barman, S., & Srinivasan, K. (2019). Ameliorative effect of zinc supplementation on compromised small intestinal health in streptozotocin-induced diabetic rats. *Chemico-biological interactions*, *307*, 37-50.

Barman, S., & Srinivasan, K. (2019). Zinc supplementation ameliorates diabetic cataract through modulation of crystallin proteins and polyol pathway in experimental rats. *Biological trace element research*, *187*(1), 212-223.

Barman, S., & Srinivasan, K. (2016). Zinc supplementation alleviates hyperglycemia and associated metabolic abnormalities in streptozotocin-induced diabetic rats. *Canadian journal of physiology and pharmacology*, *94*(12), 1356-1365.

Bolignano, D., Cernaro, V., Gembillo, G., Baggetta, R., Buemi, M., & D’Arrigo, G. (2017). Antioxidant agents for delaying diabetic kidney disease progression: a systematic review and meta-analysis. *PLoS One*, *12*(6), e0178699.

Bortolin, R. H., Abreu, B. J. D. G. A., Ururahy, M. A. G., de Souza, K. S. C., Bezerra, J. F., Loureiro, M. B., ... & Luchessi, A. D. (2015). Protection against T1DM-induced bone loss by zinc supplementation: biomechanical, histomorphometric, and molecular analyses in STZ-induced diabetic rats. *PloS one*, *10*(5), e0125349.

Capdor, J., Foster, M., Petocz, P., & Samman, S. (2013). Zinc and glycemic control: a meta-analysis of randomised placebo controlled supplementation trials in humans. *Journal of Trace Elements in Medicine and Biology*, *27*(2), 137-142.

Chimienti, F. (2013). Zinc, pancreatic islet cell function and diabetes: new insights into an old story. *Nutrition research reviews*, *26*(1), 1-11.

de Carvalho, G. B., Brandão-Lima, P. N., Maia, C. S. C., Barbosa, K. B. F., & Pires, L. V. (2017). Zinc’s role in the glycemic control of patients with type 2 diabetes: a systematic review. *Biometals*, *30*(2), 151-162.

El Dib, R., Gameiro, O. L., Ogata, M. S., Modolo, N. S., Braz, L. G., Jorge, E. C., ... & Beletate, V. (2015). Zinc supplementation for the prevention of type 2 diabetes mellitus in adults with insulin resistance. *Cochrane Database of Systematic Reviews*, (5).

Elsaed, W. M., & Mohamed, H. A. (2017). Dietary zinc modifies diabetic-induced renal pathology in rats. *Renal failure*, *39*(1), 246-257.

Ferreira, E. C., Bortolin, R. H., Freire-Neto, F. P., Souza, K. S., Bezerra, J. F., Ururahy, M. A., ... & Pedrosa, L. F. (2017). Zinc supplementation reduces RANKL/OPG ratio and prevents bone architecture alterations in ovariectomized and type 1 diabetic rats. *Nutrition research*, *40*, 48-56.

Islam, M. R., Attia, J., Ali, L., McEvoy, M., Selim, S., Sibbritt, D., & Mona, T. (2016). Zinc supplementation for improving glucose handling in pre-diabetes: a double blind randomized placebo controlled pilot study. *Diabetes research and clinical practice*, *115*, 39-46.

Jafarnejad, S., Mahboobi, S., McFarland, L. V., Taghizadeh, M., & Rahimi, F. (2019). Meta-Analysis: Effects of Zinc Supplementation Alone or with Multi-Nutrients, on Glucose Control and Lipid Levels in Patients with Type 2 Diabetes. *Preventive nutrition and food science*, *24*(1), 8.

Karamali, M., Heidarzadeh, Z., Seifati, S. M., Samimi, M., Tabassi, Z., Hajijafari, M., & Esmaillzadeh, A. (2015). Zinc supplementation and the effects on metabolic status in gestational diabetes: a randomized, double-blind, placebo-controlled trial. *Journal of Diabetes and its Complications*, *29*(8), 1314-1319.

Kibiti, C. M., & Afolayan, A. J. (2015). The biochemical role of macro and micro-minerals in the management of diabetes mellitus and its associated complications: a review. *Int J Vitam Nutr Res*, *85*, 88-103.

Kloubert, V., & Rink, L. (2015). Zinc as a micronutrient and its preventive role of oxidative damage in cells. *Food & function*, *6*(10), 3195-3204.

Liu, F., Ma, F., Kong, G., Wu, K., Deng, Z., & Wang, H. (2014). Zinc Supplementation Alleviates Diabetic Peripheral Neuropathy by Inhibiting Oxidative Stress and Upregulating Metallothionein in Peripheral Nerves of Diabetic Rats. *Biological Trace Element Research*, *158*(2), 211–218. doi: 10.1007/s12011-014-9923-9

Lobene, A. J., Kindler, J. M., Jenkins, N. T., Pollock, N. K., Laing, E. M., Grider, A., & Lewis, R. D. (2017). Zinc supplementation does not Alter indicators of insulin secretion and sensitivity in black and white female adolescents. *The Journal of nutrition*, *147*(7), 1296-1300.

Lu, Y., Liu, Y., Li, H., Wang, X., Wu, W., & Gao, L. (2015). Effect and mechanisms of zinc supplementation in protecting against diabetic cardiomyopathy in a rat model of type 2 diabetes. *Bosnian journal of basic medical sciences*, *15*(1), 14.

McCarty, M. F., & DiNicolantonio, J. J. (2015). The protection conferred by chelation therapy in post-MI diabetics might be replicated by high-dose zinc supplementation. *Medical hypotheses*, *84*(5), 451-455.

Miao, X., Sun, W., Fu, Y., Miao, L., & Cai, L. (2013). Zinc homeostasis in the metabolic syndrome and diabetes. *Frontiers of medicine*, *7*(1), 31-52.

Mishima, T., Kuroki, T., Tajima, Y., Adachi, T., Hirabaru, M., Tanaka, T., & Eguchi, S. (2014). Dietary zinc supplementation to the donor improves insulin secretion after islet transplantation in chemically induced diabetic rats. *Pancreas*, *43*(2), 236-239.

Momen‐Heravi, M., Barahimi, E., Razzaghi, R., Bahmani, F., Gilasi, H. R., & Asemi, Z. (2017). The effects of zinc supplementation on wound healing and metabolic status in patients with diabetic foot ulcer: A randomized, double‐blind, placebo‐controlled trial. *Wound Repair and Regeneration*, *25*(3), 512-520.

Pérez, A., Rojas, P., Carrasco, F., Basfi-fer, K., Pérez-Bravo, F., Codoceo, J., & Ruz, M. (2018). Zinc supplementation does not affect glucagon response to intravenous glucose and insulin infusion in patients with well-controlled type 2 diabetes. *Biological trace element research*, *185*(2), 255-261.

Ranasinghe, P., Wathurapatha, W. S., Galappatthy, P., Katulanda, P., Jayawardena, R., & Constantine, G. R. (2018). Zinc supplementation in prediabetes: A randomized double‐blind placebo‐controlled clinical trial:  *Journal of diabetes*, *10*(5), 386-397.

Ruz, M., Carrasco, F., Rojas, P., Codoceo, J., Inostroza, J., Basfi-Fer, K., & López, G. (2013). Zinc as a potential coadjuvant in therapy for type 2 diabetes. *Food and nutrition bulletin*, *34*(2), 215-221.

Ruz, M., Carrasco, F., Rojas, P., Basfi-fer, K., Hernández, M. C., & Pérez, A. (2019). Nutritional effects of zinc on metabolic syndrome and type 2 diabetes: mechanisms and main findings in human studies. *Biological trace element research*, *188*(1), 177-188.

Ruz, M., Carrasco, F., Sánchez, A., Perez, A., & Rojas, P. (2016). Does zinc really “metal” with diabetes? The epidemiologic evidence. *Current diabetes reports*, *16*(11), 111.

Sacan, O., Turkyilmaz, I. B., Bayrak, B. B., Mutlu, O., Akev, N., & Yanardag, R. (2016). Zinc supplementation ameliorates glycoprotein components and oxidative stress changes in the lung of streptozotocin diabetic rats. *Biometals*, *29*(2), 239-248.

Sadri, H., Larki, N. N., & Kolahian, S. (2017). Hypoglycemic and hypolipidemic effects of leucine, zinc, and chromium, alone and in combination, in rats with type 2 diabetes. *Biological trace element research*, *180*(2), 246-254.

Wang, S., Gu, J., Xu, Z., Zhang, Z., Bai, T., Xu, J., & Wang, Y. (2017). Zinc rescues obesity‐induced cardiac hypertrophy via stimulating metallothionein to suppress oxidative stress‐activated BCL 10/CARD 9/p38 MAPK pathway. *Journal of Cellular and Molecular Medicine*, *21*(6), 1182-1192.

Wang, S., Wang, B., Wang, Y., Tong, Q., Liu, Q., Sun, J., & Cai, L. (2017). Zinc prevents the development of diabetic cardiomyopathy in db/db mice. *International journal of molecular sciences*, *18*(3), 580.

Wang, X., Wu, W., Zheng, W., Fang, X., Chen, L., Rink, L., & Wang, F. (2019). Zinc supplementation improves glycemic control for diabetes prevention and management: a systematic review and meta-analysis of randomized controlled trials. *The American journal of clinical nutrition*.

**Amino Acids**

*Beta-alanine*

Sale, C., Artioli, G., Gualano, B., Saunders, B., Hobson, R., & Harris, R. (2013). Carnosine: from exercise performance to health. *Amino Acids*, 44(6), 1477-1491. doi: 10.1007/s00726-013-1476-2

*Leucine*

Binder, E., Bermúdez-Silva, F., André, C., Elie, M., Romero-Zerbo, S., & Leste-Lasserre, T. et al. (2013). Leucine Supplementation Protects from Insulin Resistance by Regulating Adiposity Levels. *Plos ONE*, 8(9), e74705. doi: 10.1371/journal.pone.0074705

Brunetta, H., de Camargo, C., & Nunes, E. (2018). Does l-leucine supplementation cause any effect on glucose homeostasis in rodent models of glucose intolerance? A systematic review. *Amino Acids*, 50(12), 1663-1678. doi: 10.1007/s00726-018-2658-8

Chen, K., Chen, Y., Tang, H., Hung, C., Yen, T., & Cheng, M. et al. (2018). Dietary Leucine Supplement Ameliorates Hepatic Steatosis and Diabetic Nephropathy in db/db Mice*. International Journal of Molecular Sciences*, 19(7), 1921. doi: 10.3390/ijms19071921

de Oliveira, C., Latorraca, M., de Mello, M., & Carneiro, E. (2010). Mechanisms of insulin secretion in malnutrition: modulation by amino acids in rodent models. *Amino Acids*, 40(4), 1027-1034. doi: 10.1007/s00726-010-0716-y

Kolahian, S., Sadri, H., Shahbazfar, A., Amani, M., Mazadeh, A., & Mirani, M. (2015). The Effects of Leucine, Zinc, and Chromium Supplements on Inflammatory Events of the Respiratory System in Type 2 Diabetic Rats. *PLOS ONE*, 10(7), e0133374. doi: 10.1371/journal.pone.0133374

Filiputti, E., Rafacho, A., Araújo, E., Silveira, L., Trevisan, A., & Batista, T. et al. (2010). Augmentation of insulin secretion by leucine supplementation in malnourished rats: possible involvement of the phosphatidylinositol 3-phosphate kinase/mammalian target protein of rapamycin pathway. *Metabolism*, 59(5), 635-644. doi: 10.1016/j.metabol.2009.09.007

Leenders, M., & van Loon, L. (2011). Leucine as a pharmaconutrient to prevent and treat sarcopenia and type 2 diabetes. *Nutrition Reviews*, 69(11), 675-689. doi: 10.1111/j.1753-4887.2011.00443.x

Leenders, M., Verdijk, L., van der Hoeven, L., van Kranenburg, J., Hartgens, F., & Wodzig, W. et al. (2011). Prolonged Leucine Supplementation Does Not Augment Muscle Mass or Affect Glycemic Control in Elderly Type 2 Diabetic Men. *The Journal of Nutrition*, 141(6), 1070-1076. doi: 10.3945/jn.111.138495

Macotela, Y., Emanuelli, B., Bång, A., Espinoza, D., Boucher, J., & Beebe, K. et al. (2011). Dietary Leucine - An Environmental Modifier of Insulin Resistance Acting on Multiple Levels of Metabolism. *Plos ONE*, 6(6), e21187. doi: 10.1371/journal.pone.0021187

Martins, C., Lima, V., Schoenfeld, B., & Tirapegui, J. (2017). Effects of leucine supplementation and resistance training on myopathy of diabetic rats. *Physiological Reports*, 5(10), e13273. doi: 10.14814/phy2.13273

Nairizi, A., She, P., Vary, T., & Lynch, C. (2009). Leucine Supplementation of Drinking Water Does Not Alter Susceptibility to Diet-Induced Obesity in Mice. *The Journal of Nutrition*, *139*(4), 715-719. doi: 10.3945/jn.108.100081

Pedroso, J., Zampieri, T., & Donato, J. (2015). Reviewing the Effects of l-Leucine Supplementation in the Regulation of Food Intake, Energy Balance, and Glucose Homeostasis. *Nutrients*, 7(5), 3914-3937. doi: 10.3390/nu7053914

Rachdi, L., Aiello, V., Duvillie, B., & Scharfmann, R. (2011). L-Leucine Alters Pancreatic -Cell Differentiation and Function via the mTor Signaling Pathway. *Diabetes*, 61(2), 409-417. doi: 10.2337/db11-0765

Ribeiro, H., Coqueiro, A., Lima, V., Martins, C., & Tirapegui, J. (2017). Leucine and resistance training improve hyperglycemia, white adipose tissue loss, and inflammatory parameters in an experimental model of type 1 diabetes. *Nutrition and Health*, 24(1), 19-27. doi: 10.1177/0260106017733908

Sadri, H., Larki, N., & Kolahian, S. (2017). Hypoglycemic and Hypolipidemic Effects of Leucine, Zinc, and Chromium, Alone and in Combination, in Rats with Type 2 Diabetes. *Biological Trace Element Research*, 180(2), 246-254. doi: 10.1007/s12011-017-1014-2

van Loon, L. (2012). Leucine as a pharmaconutrient in health and disease. *Current Opinion in Clinical Nutrition and Metabolic Care*, 15(1), 71-77. doi: 10.1097/mco.0b013e32834d617a

Yao, K., Duan, Y., Li, F., Tan, B., Hou, Y., Wu, G., & Yin, Y. (2016). Leucine in Obesity: Therapeutic Prospects. *Trends in Pharmacological Sciences*, 37(8), 714-727. doi: 10.1016/j.tips.2016.05.004

Zhang, Y., Guo, K., LeBlanc, R., Loh, D., Schwartz, G., & Yu, Y. (2007). Increasing Dietary Leucine Intake Reduces Diet-Induced Obesity and Improves Glucose and Cholesterol Metabolism in Mice via Multimechanisms. *Diabetes*, *56*(6), 1647-1654. doi: 10.2337/db07-012

*Taurine*

Brøns, C., Spohr, C., Storgaard, H., Dyerberg, J., & Vaag, A. (2004). Effect of taurine treatment on insulin secretion and action, and on serum lipid levels in overweight men with a genetic predisposition for type II diabetes mellitus. *European Journal Of Clinical Nutrition*, *58*(9), 1239-1247. doi: 10.1038/sj.ejcn.1601955

Chang, K. J., & Kwon, W. (2002). Immunohistochemical localization of insulin in pancreatic β-cells of taurine-supplemented or taurine-depleted diabetic rats. [*Adv Exp Med Bio*l.](https://www.ncbi.nlm.nih.gov/pubmed/11787644) 2000;483:579-87.

Chiang, S., Yeh, S., Chen, Y., Lin, S., & Tseng, J. (2014). Investigation of the Protective Effects of Taurine against Alloxan-Induced Diabetic Retinal Changes via Electroretinogram and Retinal Histology with New Zealand White Rabbits. *International Journal Of Endocrinology*, *2014*, 1-7. doi: 10.1155/2014/631549

Di Leo, M., Santini, S., Gentiloni Silveri, N., Giardina, B., Franconi, F., & Ghirlanda, G. (2004). Long-term taurine supplementation reduces mortality rate in streptozotocin-induced diabetic rats. *Amino Acids*, *27*(2), 187-191. doi: 10.1007/s00726-004-0108-2

Franconi, F., Loizzo, A., Ghirlanda, G., & Seghieri, G. (2006). Taurine supplementation and diabetes mellitus. *Current Opinion In Clinical Nutrition And Metabolic Care*, *9*(1), 32-36. doi: 10.1097/01.mco.0000196141.65362.46

Harada, N., Ninomiya, C., Osako, Y., Morishima, M., Mawatari, K., Takahashi, A., & Nakaya, Y. (2004). Taurine Alters Respiratory Gas Exchange and Nutrient Metabolism in Type 2 Diabetic Rats. *Obesity Research*, *12*(7), 1077-1084. doi: 10.1038/oby.2004.135

Hsu, Y., Yeh, S., Chen, Y., Chen, Y., Lin, S., & Tseng, J. (2012). Protective effects of taurine against alloxan-induced diabetic cataracts and refraction changes in New Zealand White rabbits. *Experimental Eye Research*, *103*, 71-77. doi: 10.1016/j.exer.2012.08.001

Kim, K., Oh, D., Kim, J., Lee, B., You, J., & Chang, K. et al. (2012). Taurine ameliorates hyperglycemia and dyslipidemia by reducing insulin resistance and leptin level in Otsuka Long-Evans Tokushima fatty (OLETF) rats with long-term diabetes. *Experimental & Molecular Medicine*, *44*(11), 665. doi: 10.3858/emm.2012.44.11.075

Moloney, M., Casey, R., O'Donnell, D., Fitzgerald, P., Thompson, C., & Bouchier-Hayes, D. (2010). Two weeks taurine supplementation reverses endothelial dysfunction in young male type 1 diabetics. *Diabetes And Vascular Disease Research*, *7*(4), 300-310. doi: 10.1177/1479164110375971

Obrosova, I. G., & Stevens, M. J. (1999). Effect of dietary taurine supplementation on GSH and NAD (P)-redox status, lipid peroxidation, and energy metabolism in diabetic precataractous lens. *Investigative ophthalmology & visual science*, *40*(3), 680-688.

Ribeiro, R., Bonfleur, M., Amaral, A., Vanzela, E., Rocco, S., Boschero, A., & Carneiro, E. (2009). Taurine supplementation enhances nutrient-induced insulin secretion in pancreatic mice islets. *Diabetes/Metabolism Research And Reviews*, *25*(4), 370-379. doi: 10.1002/dmrr.959

Sirdah, M. (2015). Protective and therapeutic effectiveness of taurine in diabetes mellitus: A rationale for antioxidant supplementation. *Diabetes & Metabolic Syndrome: Clinical Research & Reviews*, *9*(1), 55-64. doi: 10.1016/j.dsx.2014.05.001

Spohr, C., BrØns, C., Winther, K., Dyerberg, J., & Vaag, A. (2005). No effect of taurine on platelet aggregation in men with a predisposition to type 2 diabetes mellitus. *Platelets*, *16*(5), 301-305. doi: 10.1080/09537100400020575

Wang, L., Yu, Y., Zhang, L., Wang, Y., Niu, N., Li, Q., & Guo, L. (2008). Taurine rescues vascular endothelial dysfunction in streptozocin-induced diabetic rats: Correlated with downregulation of LOX-1 and ICAM-1 expression on aortas. *European Journal Of Pharmacology*, *597*(1-3), 75-80. doi: 10.1016/j.ejphar.2008.08.031

Yao, H., Lin, P., Chang, Y., Chen, C., Chiang, M., & Chang, L. et al. (2009). Effect of taurine supplementation on cytochrome P450 2E1 and oxidative stress in the liver and kidneys of rats with streptozotocin-induced diabetes. *Food And Chemical Toxicology*, *47*(7), 1703-1709. doi: 10.1016/j.fct.2009.04.030

You, J. S., & Chang, K. J. (1998). Effects of taurine supplementation on lipid peroxidation, blood glucose and blood lipid metabolism in streptozotocin-induced diabetic rats. In *Taurine 3* (pp. 163-168). Springer, Boston, MA.

Yu, X., Xu, Z., Mi, M., Xu, H., Zhu, J., & Wei, N. et al. (2007). Dietary Taurine Supplementation Ameliorates Diabetic Retinopathy via Anti-excitotoxicity of Glutamate in Streptozotocin-induced Sprague-Dawley Rats. *Neurochemical Research*, *33*(3), 500-507. doi: 10.1007/s11064-007-9465-z

**Other**

*Caffeine*

Hamed, E. (2018). Caffeine Toxicity Following Ingestion of an Exercise Supplement by a Patient with Type 1 Diabetes. *European Journal Of Case Reports In Internal Medicine*, (Vol 5 No 10), 1. doi: 10.12890/2018_000957

Stefanello, N., Schmatz, R., Pereira, L., Cardoso, A., Passamonti, S., & Spanevello, R. et al. (2016). Effects of chlorogenic acid, caffeine and coffee on components of the purinergic system of streptozotocin-induced diabetic rats. *The Journal of Nutritional Biochemistry*, *38*, 145-153. doi: 10.1016/j.jnutbio.2016.08.015

Zaharieva, D., Miadovnik, L., Rowan, C., Gumieniak, R., Jamnik, V., & Riddell, M. (2015). Effects of acute caffeine supplementation on reducing exercise-associated hypoglycaemia in individuals with Type 1 diabetes mellitus. *Diabetic Medicine*, *33*(4), 488-496. doi: 10.1111/dme.12857

*Dietary Fiber*

Andrade, E., Lima, A., Nunes, I., Orlando, D., Gondim, P., & Zangeronimo, M. et al. (2016). Exercise and Beta-Glucan Consumption (Saccharomyces cerevisiae) Improve the Metabolic Profile and Reduce the Atherogenic Index in Type 2 Diabetic Rats (HFD/STZ). *Nutrients*, *8*(12), 792. doi: 10.3390/nu8120792

Barengolts, E. (2016). GUT MICROBIOTA, PREBIOTICS, PROBIOTICS, AND SYNBIOTICS IN MANAGEMENT OF OBESITY AND PREDIABETES: REVIEW OF RANDOMIZED CONTROLLED TRIALS. *Endocrine Practice*, *22*(10), 1224-1234. doi: 10.4158/ep151157.ra

Canfora, E., van der Beek, C., Hermes, G., Goossens, G., Jocken, J., & Holst, J. et al. (2017). Supplementation of Diet With Galacto-oligosaccharides Increases Bifidobacteria, but Not Insulin Sensitivity, in Obese Prediabetic Individuals. *Gastroenterology*, *153*(1), 87-97.e3. doi: 10.1053/j.gastro.2017.03.051

Chappuis, E., Morel-Depeisse, F., Bariohay, B., & Roux, J. (2017). Alpha-Galacto-Oligosaccharides at Low Dose Improve Liver Steatosis in a High-Fat Diet Mouse Model. *Molecules*, *22*(10), 1725. doi: 10.3390/molecules22101725

Chung, P., Wu, Y., Chen, P., Fung, C., Hsu, C., & Chen, L. (2016). Lactobacillus salivarius reverse diabetes-induced intestinal defense impairment in mice through non-defensin protein. *The Journal Of Nutritional Biochemistry*, *35*, 48-57. doi: 10.1016/j.jnutbio.2016.05.013

de Carvalho, C., de Paula, T., Viana, L., Machado, V., de Almeida, J., & Azevedo, M. (2017). Plasma glucose and insulin responses after consumption of breakfasts with different sources of soluble fiber in type 2 diabetes patients: a randomized crossover clinical trial. *The American Journal Of Clinical Nutrition*, ajcn157263. doi: 10.3945/ajcn.117.157263

Farhangi, M. A., Javid, A. Z., & Dehghan, P. (2016). The effect of enriched chicory inulin on liver enzymes, calcium homeostasis and hematological parameters in patients with type 2 diabetes mellitus: A randomized placebo-controlled trial. *Primary care diabetes*, *10*(4), 265-271.

Florowska, A., Krygier, K., Florowski, T., & Dłużewska, E. (2016). Prebiotics as functional food ingredients preventing diet-related diseases. *Food & Function*, *7*(5), 2147-2155. doi: 10.1039/c5fo01459j

Garcia-Mazcorro, J., Mills, D., Murphy, K., & Noratto, G. (2017). Effect of barley supplementation on the fecal microbiota, caecal biochemistry, and key biomarkers of obesity and inflammation in obese db/db mice. *European Journal Of Nutrition*, *57*(7), 2513-2528. doi: 10.1007/s00394-017-1523-y

Gonai, M., Shigehisa, A., Kigawa, I., Kurasaki, K., Chonan, O., & Matsuki, T. et al. (2017). Galacto-oligosaccharides ameliorate dysbiotic Bifidobacteriaceae decline in Japanese patients with type 2 diabetes. *Beneficial Microbes*, *8*(5), 705-716. doi: 10.3920/bm2016.0230

Honsek, C., Kabisch, S., Kemper, M., Gerbracht, C., Arafat, A., & Birkenfeld, A. et al. (2018). Fibre supplementation for the prevention of type 2 diabetes and improvement of glucose metabolism: the randomised controlled Optimal Fibre Trial (OptiFiT). *Diabetologia*, *61*(6), 1295-1305. doi: 10.1007/s00125-018-4582-6

Jane, M., McKay, J., & Pal, S. (2019). Effects of daily consumption of psyllium, oat bran and polyGlycopleX on obesity-related disease risk factors: A critical review. *Nutrition*, *57*, 84-91. doi: 10.1016/j.nut.2018.05.036

Jovanovski, E., Khayyat, R., Zurbau, A., Komishon, A., Mazhar, N., & Sievenpiper, J. et al. (2019). Should Viscous Fiber Supplements Be Considered in Diabetes Control? Results From a Systematic Review and Meta-analysis of Randomized Controlled Trials. *Diabetes Care*, *42*(5), 755-766. doi: 10.2337/dc18-1126

Kim, Y., Keogh, J., & Clifton, P. (2017). Probiotics, prebiotics, synbiotics and insulin sensitivity. *Nutrition Research Reviews*, *31*(1), 35-51. doi: 10.1017/s095442241700018x

Marques, F., Nelson, E., Chu, P., Horlock, D., Fiedler, A., & Ziemann, M. et al. (2017). High-Fiber Diet and Acetate Supplementation Change the Gut Microbiota and Prevent the Development of Hypertension and Heart Failure in Hypertensive Mice. *Circulation*, *135*(10), 964-977. doi: 10.1161/circulationaha.116.024545

Neyrinck, A., Hiel, S., Bouzin, C., Campayo, V., Cani, P., Bindels, L., & Delzenne, N. (2018). Wheat-derived arabinoxylan oligosaccharides with bifidogenic properties abolishes metabolic disorders induced by western diet in mice. *Nutrition & Diabetes*, *8*(1). doi: 10.1038/s41387-018-0019-z

O’Connor, S., Chouinard-Castonguay, S., Gagnon, C., & Rudkowska, I. (2017). Prebiotics in the management of components of the metabolic syndrome. *Maturitas*, *104*, 11-18. doi: 10.1016/j.maturitas.2017.07.005

Okubo, H., Nakatsu, Y., Kushiyama, A., Yamamotoya, T., Matsunaga, Y., & Inoue, M. et al. (2018). Gut Microbiota as a Therapeutic Target for Metabolic Disorders. *Current Medicinal Chemistry*, *25*(9), 984-1001. doi: 10.2174/0929867324666171009121702

Pedersen, C., Gallagher, E., Horton, F., Ellis, R., Ijaz, U., & Wu, H. et al. (2016). Host–microbiome interactions in human type 2 diabetes following prebiotic fibre (galacto-oligosaccharide) intake. *British Journal Of Nutrition*, *116*(11), 1869-1877. doi: 10.1017/s0007114516004086

Stahel, P., Kim, J., Xiao, C., & Cant, J. (2017). Of the milk sugars, galactose, but not prebiotic galacto-oligosaccharide, improves insulin sensitivity in male Sprague-Dawley rats. *PLOS ONE*, *12*(2), e0172260. doi: 10.1371/journal.pone.0172260

Steinert, R., Raederstorff, D., & Wolever, T. (2016). Effect of Consuming Oat Bran Mixed in Water before a Meal on Glycemic Responses in Healthy Humans—A Pilot Study. *Nutrients*, *8*(9), 524. doi: 10.3390/nu8090524

Vallianou, N., Stratigou, T., & Tsagarakis, S. (2018). Microbiome and diabetes: Where are we now?. *Diabetes Research And Clinical Practice*, *146*, 111-118. doi: 10.1016/j.diabres.2018.10.008

Wu, T., Lin, C., Chang, C., Lin, T., Martel, J., & Ko, Y. et al. (2018). Gut commensal Parabacteroides goldsteinii plays a predominant role in the anti-obesity effects of polysaccharides isolated from Hirsutella sinensis. *Gut*, *68*(2), 248-262. doi: 10.1136/gutjnl-2017-315458

Yoo, J., & Kim, S. (2016). Probiotics and Prebiotics: Present Status and Future Perspectives on Metabolic Disorders. *Nutrients*, *8*(3), 173. doi: 10.3390/nu8030173

Zheng, J., Li, H., Zhang, X., Jiang, M., Luo, C., & Lu, Z. et al. (2018). Prebiotic Mannan-Oligosaccharides Augment the Hypoglycemic Effects of Metformin in Correlation with Modulating Gut Microbiota. *Journal Of Agricultural And Food Chemistry*, *66*(23), 5821-5831. doi: 10.1021/acs.jafc.8b00829

*Protein*

Baye, E., Ukropec, J., de Courten, M., Mousa, A., Kurdiova, T., & Johnson, J. et al. (2018). Carnosine Supplementation Improves Serum Resistin Concentrations in Overweight or Obese Otherwise Healthy Adults: A Pilot Randomized Trial. *Nutrients*, *10*(9), 1258. doi: 10.3390/nu10091258

Guo, Q., Hu, H., Zhou, Y., Yan, Y., Wei, X., & Fan, X. et al. (2019). Glucosamine induces increased musclin gene expression through endoplasmic reticulum stress-induced unfolding protein response signaling pathways in mouse skeletal muscle cells. *Food And Chemical Toxicology*, *125*, 95-105. doi: 10.1016/j.fct.2018.12.051

Hidayat, K., Du, X., & Shi, B. (2019). Milk in the prevention and management of type 2 diabetes: The potential role of milk proteins. *Diabetes/Metabolism Research And Reviews*. doi: 10.1002/dmrr.3187

Kawabeta, K., Hase-Tamaru, S., Yuasa, M., Suruga, K., Sugano, M., & Koba, K. (2019). Dietary β-Conglycinin Modulates Insulin Sensitivity, Body Fat Mass, and Lipid Metabolism in Obese Otsuka Long-Evans Tokushima Fatty (OLETF) Rats. *Journal Of Oleo Science*, *68*(4), 339-350. doi: 10.5650/jos.ess18232

*Trans Fat*

Diane, A., Borthwick, F., Mapiye, C., Vahmani, P., David, R., & Vine, D. et al. (2016). Beef Fat Enriched with Polyunsaturated Fatty Acid Biohydrogenation Products Improves Insulin Sensitivity Without Altering Dyslipidemia in Insulin Resistant JCR:LA-cp Rats. *Lipids*, *51*(7), 821-831. doi: 10.1007/s11745-016-4148-7

Schmidt J, Liebscher K, Merten N, Grundmann M, Mielenz M, Sauerwein H, Christiansen E, Due-Hansen ME, Ulven T, Ullrich S, Gomeza J, Drewke C, Kostenis E. (2011). Conjugated Linoleic Acids Mediate Insulin Release through Islet G Protein-coupled Receptor FFA1/GPR40. *Journal of Biological Chemistry*, *286*, 11890-11894.

McCrorie, T., Keaveney, E., Wallace, J., Binns, N., & Livingstone, M. (2011). Human health effects of conjugated linoleic acid from milk and supplements. *Nutrition Research Reviews*, *24*(2), 206-227. doi: 10.1017/s0954422411000114

McFarlin, B., Strohacker, K., & Kueht, M. (2008). Pomegranate seed oil consumption during a period of high-fat feeding reduces weight gain and reduces type 2 diabetes risk in CD-1 mice. *British Journal Of Nutrition*, *102*(1), 54-59. doi: 10.1017/s0007114508159001

Riserus, U., Vessby, B., Arner, P., & Zethelius, B. (2004). Supplementation with trans10cis12-conjugated linoleic acid induces hyperproinsulinaemia in obese men: close association with impaired insulin sensitivity. *Diabetologia*, *47*(6). doi: 10.1007/s00125-004-1421-8

Wang, X., & Chan, C. (2014). Trans-11 Vaccenic Acid Improves Glucose Turnover In Vivo and Insulin Secretion In Vitro in Models of Type 2 Diabetes. *Canadian Journal Of Diabetes*, *38*(5), S17. doi: 10.1016/j.jcjd.2014.07.045
